# Microstructural and Surface Energy Evaluation of Concrete Coatings Modified with Methyl Ester and Stearic Acid

**DOI:** 10.3390/ma19163493

**Published:** 2026-08-18

**Authors:** Robert Hunek, Martyna Janek, Wojciech Franus

**Affiliations:** 1 Doctoral School, Lublin University of Technology Nadbystrzycka 38B/406, 20-618 Lublin, Poland; d597@pollub.edu.pl; 2 Faculty of Civil Engineering and Architecture, Lublin University of Technology, Nadbystrzycka 40, 20-618 Lublin, Poland; m.janek@pollub.pl

**Keywords:** concrete, methyl ester, stearic acid, coating, contact angle, microstructure

## Abstract

The purpose of this paper is to determine the influence of the stearic acid and methyl esters on the properties of protective coatings applied to reinforced concrete objects. The evaluation of protective effectiveness included compositional analysis (XRD, XRF), surface properties (contact angle, surface energy, UV resistance), soiling resistance, roughness, and surface morphology (SEM). The scanning microscopy SEM images showed a relatively uniform distribution of resin on the concrete surface. The lateral dimensions of the epoxy-rich surface domains ranged from 8 μm to 40 μm. All analysed samples exhibited a very similar chemical composition, in which SiO_2_ was the dominant component. In situ tests were conducted on an operating reinforced-concrete cooling tower. Among the investigated coating systems, ER1 + SA exhibited the highest observed mean apparent static water contact angle (CA) of 131°. A strong inverse empirical relationship was observed between the apparent contact angle and average surface roughness. UV ageing caused small numerical decreases in contact angle for most systems. After one year of operation, the ER2 epoxy coating modified with methyl esters was the best-preserved of the analysed variants.

## 1. Introduction

The degradation of concrete and concrete structures constitutes one of the key research issues in the field of construction materials. Cement-based materials are porous and hydrophilic, which facilitates the ingress of water and aggressive agents such as carbon dioxide, chlorides, and sulphates. These processes are major causes of concrete deterioration, including the leaching of calcium compounds, carbonation, and reinforcement corrosion [1,2]. Such processes lead to a gradual deterioration of the material’s functional properties and a reduction in the service life of structures. Accordingly, the mechanisms of concrete degradation remain the subject of intensive research due to their significant impact on the long-term performance of engineering structures.

Various approaches are employed to mitigate the degradation of cement-based composites, including modifications to the mix composition and the densification of the material structure [3]. Particular importance is attributed to surface protection systems [4,5], which are among the most widely applied methods under service conditions. The general mechanism of concrete protection through surface treatments is based on limiting the ingress of aggressive substances by forming a tight barrier layer that separates the external environment from the material structure [6]. Pan et al. [7] classified the main types and mechanisms of protective coatings and concrete surface treatments into four groups, depending on their mode of interaction with the substrate and their functional role. Surface coatings (i) form a continuous polymer layer that acts as a physical barrier, restricting the ingress of aggressive agents. Their effectiveness depends on the microstructure of the material, the degree of cross-linking, and, particularly in nanocomposites, the increased tortuosity of diffusion pathways [8,9]. Hydrophobic impregnation (ii) involves the penetration of compounds, primarily silanes and siloxanes, into the concrete pore system. This modifies the wettability of the material, thereby reducing the transport of water and dissolved substances while maintaining vapour permeability [10]. Pore-blocking treatments (iii) are based on the formation of insoluble products, mainly through chemical reactions, which fill the capillary structure and reduce material permeability [11]. Multifunctional surface treatments (iv) combine several mechanisms of action [12,13], for example by integrating a hydrophobic effect with microstructural modification through the formation of silica gel and additional C-S-H phases resulting from interactions with the cement matrix.

Concrete surface-protection materials include conventional polymer coatings (epoxy, acrylic, and polyurethane), polymer nanocomposite coatings, as well as cement-based coatings, including polymer-modified and geopolymer systems [7]. Among these materials, epoxy resins are widely used due to their high degree of cross-linking, which enables the formation of a dense barrier limiting the ingress of water, chloride ions, and other aggressive agents. In addition, they exhibit good adhesion to the concrete substrate [14] and favourable mechanical performance [15]. Pan et al. [16] reported that epoxy coatings demonstrate the highest abrasion resistance among organic coatings. Acrylic coatings, based on acrylic polymers (derivatives of acrylic acid), are valued for their resistance to weathering, oxidation, and alkaline environments. They form a relatively tight protective layer that limits water ingress and carbonation under standard service conditions. However, their adhesion and deformability are generally lower than those of epoxy resins, and their effectiveness in reducing water penetration, particularly in highly porous concrete, may be inferior to that of silane- or polyurethane-based systems [17]. Polymer–clay nanocomposites are advanced two-phase materials consisting of a polymer matrix in which nanoscale inorganic particles, most commonly layered silicates, are dispersed. The presence of impermeable platelets increases the diffusion path for gases, resulting in a significant reduction in material permeability even at low filler contents [18]. Maksimov et al. [19] investigated nanocomposites based on a styrene–acrylic copolymer (SAC) filled with natural, unmodified montmorillonite (MMT) to assess their moisture barrier properties. They demonstrated that the incorporation of as little as 3 wt.% MMT led to a 2.3-fold reduction in the water vapour permeability coefficient, attributed to the substantial elongation of the diffusion pathway of water molecules through the platelet-shaped filler particles.

Hydrophobic impregnation is used to impart water repellency to concrete surfaces, commonly indicated by an apparent water contact angle exceeding 90° [7]. The most commonly used materials in hydrophobisation technology are silanes and siloxanes, which contain alkyl and alkoxy groups responsible for reducing surface tension and enabling effective bonding of the hydrophobic agent to the concrete substrate, respectively [20,21]. Numerous studies have shown that the application of silanes effectively reduces the water absorption of concrete, which in turn limits chloride ingress and lowers the risk of reinforcement corrosion [22]. Importantly, their protective performance has been confirmed not only in the short term but also under long-term service conditions, demonstrating the durability of the hydrophobic effect and the sustained reduction in the transport of water and aggressive ions over time. Christodoulou et al. [23] demonstrated that silane-based impregnation can provide a durable residual protective effect even after 20 years of service. The analysis of cores extracted from real bridge elements confirmed the persistence of hydrophobic properties and a statistically significant reduction in water absorption compared with reference samples, indicating the long-term effectiveness of this method despite gradual degradation over time.

Recent research has increasingly focused on the modification of hydrophobic coatings to develop materials with enhanced protective performance and improved durability compared with conventional impregnation systems [24]. Ajorloo et al. [25] incorporated stearic acid together with SiO_2_ nanoparticles, introduced to increase surface roughness, which resulted in enhanced hydrophobicity. The effectiveness of this approach was confirmed through a range of physicochemical, mechanical, and functional tests. These included, among others, a significant reduction in water absorption of up to 99.6%, a high contact angle (approximately 137°), and good durability under UV radiation, with a decrease of approximately 10% in WCA. In a study by Zhao et al. [26], a multilayer superhydrophobic coating was developed, incorporating a micro- and nanostructured rough layer based on diamond micropowder and nanosilica, along with an epoxy resin (E51) modified with polytetrafluoroethylene as a low-surface-energy component. The proposed system exhibited high hydrophobicity and improved resistance to abrasion, erosion, and environmental exposure compared with conventional silane-based impregnation systems.

Despite extensive research on concrete surface protection systems, a clear research gap remains in the comprehensive and comparative evaluation of different types of protection, taking into account not only surface properties and durability under ageing conditions, but also their actual performance under service conditions. In particular, studies simultaneously addressing conventional polymer coatings and hydrophobic impregnation systems, together with their modifications leading to multifunctional behaviour, remain limited.

The present study aims to provide a comprehensive assessment of the effectiveness of selected concrete surface-protection systems representing different mechanisms of action, including polymer coatings and hydrophobic impregnation, and their modification with fatty-acid-derived organic additives. The analysed systems represent various protection strategies, encompassing both surface coatings and hydrophobic treatments. Through modification with organic additives, the investigated coatings exhibit characteristics of multifunctional materials, combining barrier properties with enhanced surface performance. The experimental programme included the preparation of C16/20 concrete and the application of five selected commercial surface-protection products (silane-, epoxy-, methacrylate-, and silicic-acid-ester-based), including resins modified with stearic acid or methyl esters. The evaluation of protective performance comprised compositional analysis (XRD, XRF), surface properties (contact angle, surface energy, UV resistance), soiling resistance, surface roughness, and surface morphology (SEM).

## 2. Materials and Methods

### 2.1. Materials

A C16/20 concrete mix was designed [27]. The mix proportions were as follows: Portland cement CEM I 42.5R—350 kg/m^3^, sand (0/4 mm)—719 kg/m^3^, gravel (4/16 mm)—1186 kg/m^3^, and water—155 L/m^3^.

The water-to-cement ratio (*w*/*c*) was set at 0.4.

**Figure 1:**
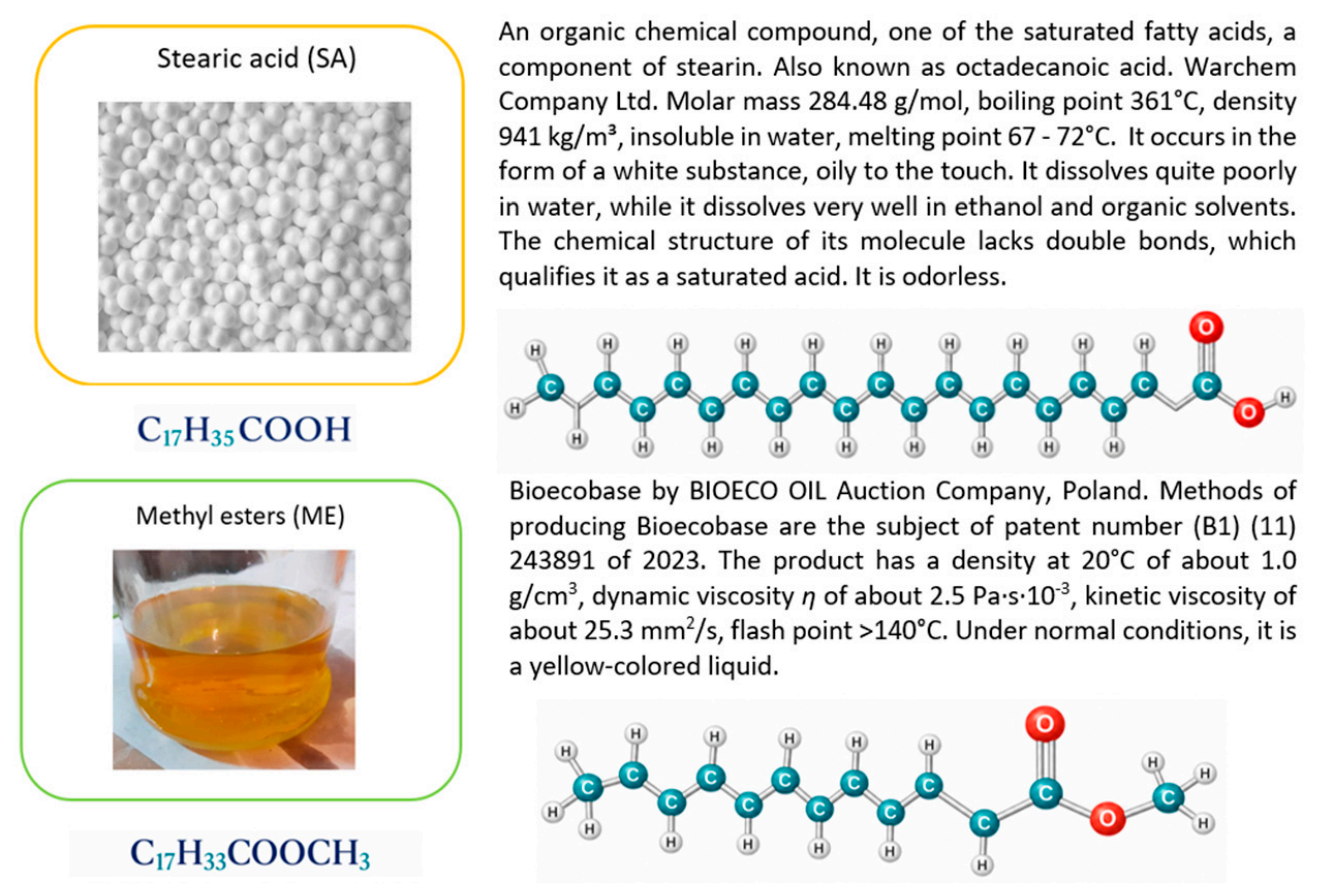
Fatty-acid-derived modifiers used in the study (authors’ illustration and photographs).

Portland cement CEM I 42.5 R (Cemex Poland, Chełm Cement plant, Chełm, Poland) was used as a binder. The cement complied with the requirements of the following standard: PN-EN 197-1:2012 [28]. The physical properties of the cement were as follows: specific surface area—3.42 cm^2^/g, water demand—27.3%, compressive strength after 2 days—28.8 MPa, compressive strength after 28 days—54.1 MPa, initial setting time—146 min, setting time—190 min, and density—3.09 g/cm^3^ [29]. Natural rinsed quartz sand and natural gravel were used for concrete preparation. Five representative commercial surface-protection products produced by established manufacturers were chosen based on prior experience and initial laboratory evaluations (Table 1). The modifiers are shown in Figure 1.

In the present study, a 20 vol.% fraction of stearic acid (SA) or methyl esters (MEs) was selected on the basis of preliminary investigations showing a marked hydrophobic effect and improved coating surface properties while maintaining adequate cohesion and adhesion to concrete. Dosages above 20%, particularly for stearic acid, adversely affected certain resin properties, including adhesion and workability; rapid hardening was observed for SA. The concentration of modifiers was not fully optimised, as the objective of the study was to assess the effectiveness of a practically feasible and economically viable dosage that would not compromise the mechanical and adhesive performance of the resins. A SONICS CVX750 ultrasonic homogenizer (Sonics & Materials, Inc., Newtown, CT, USA) equipped with an H22 sonotrode with a tip diameter of 22 mm, a maximum amplitude of 100 μm and a sound pressure density of 85 W/cm^2^ was used for the sonication process. Sonication lasted 7 min: the amplitude was 20% for the first 30 s and 80% for the remaining time. In total, 16 distinct variants were evaluated.

**Figure 2:**
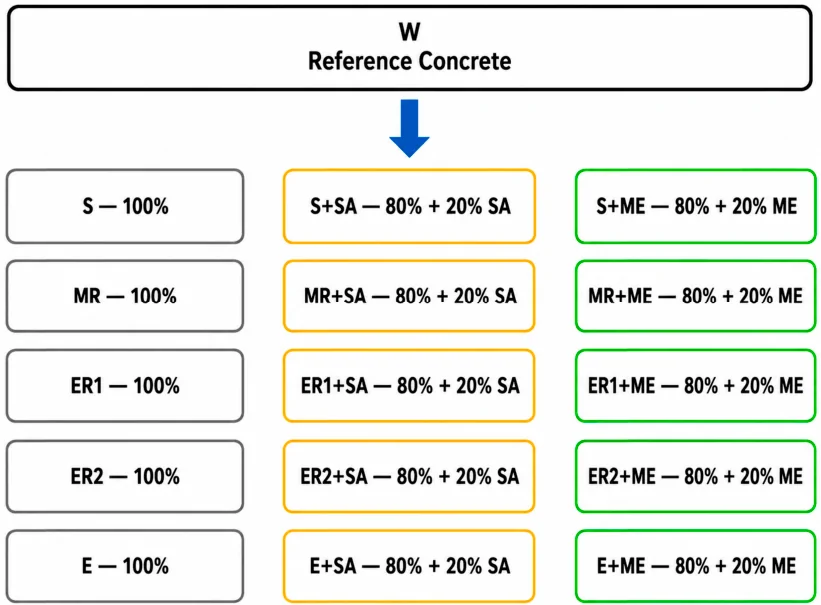
Block diagram of resin-modified concrete.

Figure 2 shows a block diagram of resin-modified concrete.

Concrete of strength class C16/20 was cast in two layers into 150 × 150 × 150 mm specimens.

Each mould was filled to approximately half its height and compacted on a vibrating table for 1 min. The remaining concrete was then added and compacted for a further 1 min.

Specimens were stored and cured in accordance with EN 12390–2 [30]. After 28 days of curing, once the target strength had been achieved, the samples were dried to constant mass at 100 °C. After cooling to ambient temperature, resin coatings were applied using a brush in two layers, following the manufacturers’ guidelines.

The present investigation is complementary to the research programme previously reported in Reference [31]. The same concrete composition, coating families, modifier types and dosages, and preparation procedures were used to enable evaluation of the same material systems using different and complementary methods. Reference [31] focused on rheological properties and macroscopic durability indicators, including water absorptivity, watertightness, water-vapour diffusion, freeze–thaw resistance, and resistance to salt crystallisation. The present study focuses on surface wettability, surface free energy, UV resistance, surface contamination, roughness, plan-view SEM/EDS characteristics, and one-year in situ performance. Water absorptivity was not re-investigated as a new experimental variable in the present study.

### 2.2. Methods

#### 2.2.1. X-Ray Diffraction (XRD)

The phase composition of powdered coated-concrete samples was analysed by X-ray powder diffraction (XRD) using a Panalytical X’pert APD X-ray diffractometer equipped with a PW 3020 goniometer, Cu X-ray tube and graphite monochromator. The diffraction patterns were analysed using X’Pert HighScore 3.0 software, with phase identification performed using the PDF-2 (2010) database of the JCPDS-ICDD.

#### 2.2.2. X-Ray Fluorescence (XRF)

X-ray fluorescence (XRF) was used to determine the chemical composition of the concrete samples with coatings. Measurements were performed using a Panalytical Epsilon 3 spectrometer equipped with a Rh X-ray tube (9 W, 50 kV, 1 mA), a 4096-channel spectrum analyser, six measurement filters (Cu-300, Cu-500, Al-50, Al-200, Ti, Ag), and a high-resolution SDD detector cooled with a Peltier element. The analysis was carried out on powdered samples taken from the near-surface layer of the coated concrete (the coating together with the adjacent concrete layer).

#### 2.2.3. Apparent Static Water Contact (CA) and Surface Free Energy (SFE) Before and After UV Ageing

Apparent static water contact angle measurements were performed using an OCA 25 optical goniometer (DataPhysics Instruments, Filderstadt, Germany) equipped with a digital camera and image-analysis software. Three independent specimens measuring 50 × 50 × 2.5 mm were examined for each treatment system. Before testing, the specimens were conditioned under laboratory conditions for 24 h, washed with deionised water, dried at 60 ± 5 °C to constant mass, and cooled to 22 ± 1 °C.

Six distilled-water droplets, each with a volume of 1.5 μL, were deposited using a micropipette at spatially separated locations on each specimen. Droplets were placed away from specimen edges and macroscopically visible surface defects. Images were acquired at 25 frames per second, and the apparent static contact angle was determined 2 s after droplet deposition using the tangent method. The values obtained for the six droplets were averaged to produce one specimen-level result. The same procedure and evaluation time were applied before and after UV ageing.

The apparent droplet behaviour during the measurement interval was also visually monitored. No visible change in droplet base diameter or volume was detected on the coated surfaces during this interval. The reference concrete showed visible spreading and partial absorption.

Prior to testing, all samples were conditioned in the laboratory for 24 h to stabilise their surface free energy. Subsequently, the samples were washed with deionised water, dried at 60 ± 5 °C to constant mass, and cooled to room temperature. Initial measurements of contact angle and SFE were performed before ageing. The samples were then subjected to accelerated UV ageing, after which the measurements were repeated under identical conditions.

#### 2.2.4. UV Ageing

Ultraviolet radiation has sufficient photon energy to break chemical bonds This causes organic coating materials to partially break their chemical bonds after absorbing UV light energy, thereby initiating photolysis and photo-oxidation. UV exposure may also weaken the coating–substrate interface and reduce surface hydrophobicity. The UV resistance of the coatings was evaluated in a TestAn Xentest 2200 xenon-arc ageing chamber.

The chamber settings were selected to simulate one year of outdoor solar exposure under the average annual UV irradiance conditions in Poland. The test parameters were as follows: exposure time, 720 h; chamber temperature, 63 °C; and irradiance, 0.51 W/m^2^. The specimens previously used for contact angle measurements were exposed and after the test was completed, the samples were reexamined in the goniometer.

#### 2.2.5. Resistance to Dirt (Ink and Coal Dust)

The protective systems were applied to concrete specimens measuring 50 × 50 × 20 mm. After curing for one week, their resistance to two contaminants, ink and coal dust, was assessed. The assessment recorded whether the contaminants remained on the surface, penetrated the material, or were repelled. For ink, spreading and runoff were also observed; for coal dust, particle adhesion was assessed. These observations enabled a comparative evaluation of the soiling resistance of the coated concrete.

#### 2.2.6. Surface Roughness (Optical Microscopy)

Surface roughness was evaluated in accordance with the British standard BS EN ISO 4287:1998+A1:2009 [32]. Surface roughness measurements were performed using a Keyence VHX-7000 optical microscope equipped with a VHX-E20 lens. Surface roughness was evaluated using a Keyence VHX-7000 digital optical microscope equipped with a VHX-E20 lens. For each treatment system, three independent specimens were examined. On each specimen, three independent roughness profiles were acquired from spatially separated surface regions selected away from specimen edges and macroscopically visible defects.

Before calculating the roughness parameters, the profiles were levelled by removing the overall surface inclination and processed using a Gaussian filter with a short-wavelength cut-off (λs) of 20 μm and a long-wavelength cut-off (λc) of 0.1 mm.

The reported values represent the mean of 3 profiles × 3 specimens, and the corresponding standard deviations have now been added. For each treatment system, the reported result is the mean of nine profiles measured on three independent specimens. Variability is reported as the standard deviation.

**Figure 3:**
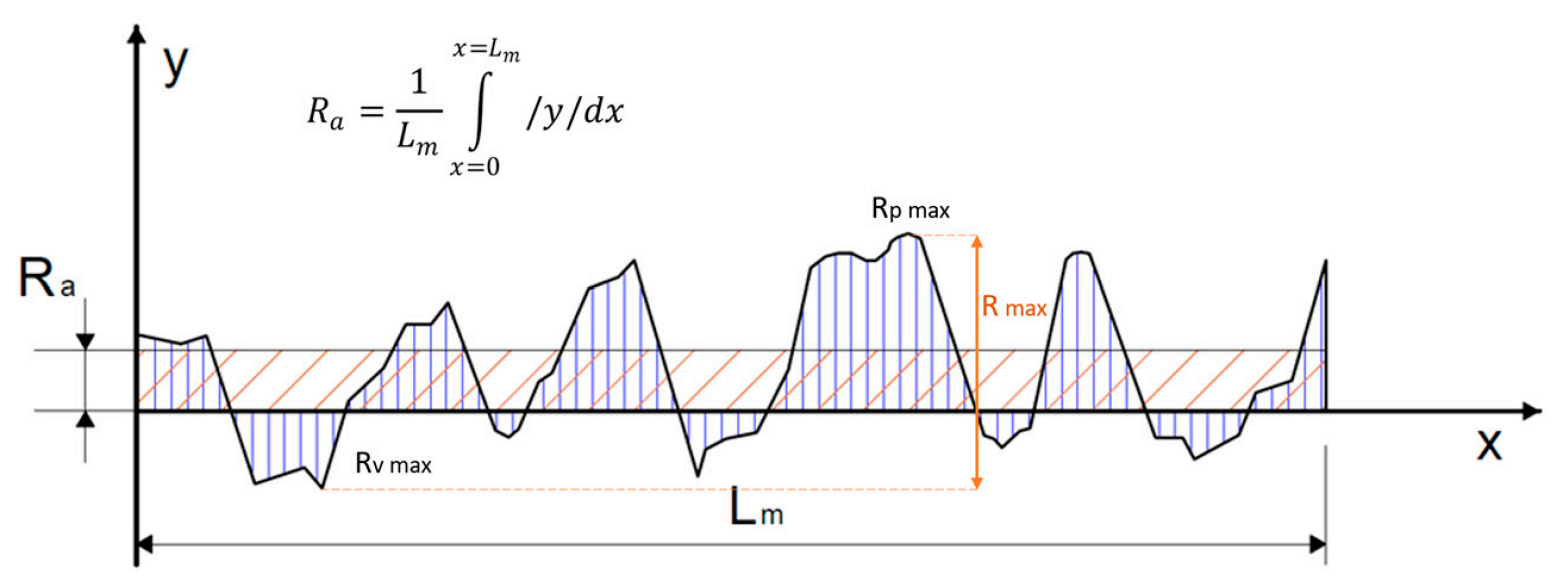
Schematic diagram of roughness curve parameters.

The analysis was carried out using a graphical user interface (GUI), which enabled the calculation of roughness profile parameters and the evaluation of geometric features, including distances, maximum peak height (*R_p_*), and maximum valley depth (*R_v_*). For the characterisation of coating surface roughness, the arithmetic mean roughness (*R_a_*) and the maximum height (*R_max_*) were selected as the primary parameters (Figure 3). Due to its heterogeneous nature, concrete exhibits a slightly irregular surface, which can be described using the following parameters:

*R_a_*—arithmetic mean roughness; *R_p_*—maximum peak height; *R_v_*_—_maximum valley depth; *R_max_*—total height of the profile.

*R_max_* = *R_v_* + *R_p_*

#### 2.2.7. Microstructural Analysis by Scanning Electron Microscopy (SEM)

The morphology and microstructure of the coatings were examined by scanning electron microscopy (SEM) using an FEG Quanta 250 microscope equipped with an EDAX energy-dispersive X-ray spectroscopy (EDS) system for elemental analysis. The specimens were mounted on holders using carbon tape.

#### 2.2.8. In Situ Assessment

Based on the results of the laboratory analyses, three modified resin-based coatings (ER2 + ME, ER1 + SA, ER1 + ME) were selected for field assessment. Subsequently, selected sections of the internal walls of a cooling tower at PGE Energia Ciepła S.A. in Lublin were coated with these systems in September 2024. After the coatings had fully cured, their initial condition was documented. The field exposure was conducted on selected areas of the internal reinforced-concrete walls of an operating cooling tower. The exposure conditions differed substantially from those of an external façade. The investigated surfaces were sheltered from direct rainfall and received only limited indirect or diffuse solar radiation. They were not exposed to seawater, de-icing salts, or chloride-containing process water.

The dominant environmental exposure was prolonged and repeated contact with deionised process water through runoff, splashing, condensation, and water aerosol. Relative humidity inside the tower was very high during operation, while operational shutdowns and maintenance generated intermittent wetting–drying periods. Seasonal temperature variations were characteristic of the Central and Eastern European climate, although the coating-surface temperature was additionally influenced by tower operation, evaporative cooling, and contact with process water.

Continuous monitoring of surface temperature, relative humidity, UV irradiance, and freeze–thaw events was not performed. Therefore, the field assessment represents a qualitative evaluation under actual service conditions rather than a controlled exposure test with a quantified environmental dose. The cooling tower was then operated under normal conditions for one year. After one year of service, the condition of the coatings on the cooling tower walls was evaluated in situ. The walls were subsequently cleaned using a high-pressure washer, and the coatings was reassessed after cleaning.

#### 2.2.9. Statistical Analysis

All variables were summarised using descriptive statistics and box-and-whisker plots. The Kruskal–Wallis [33] test was used to evaluate overall differences among the 16 treatment systems. When the omnibus test was significant, Dunn’s post hoc test [34] with correction for multiple comparisons was applied. Owing to the limited number of independent specimens per treatment system (*n* = 3), the post hoc results were interpreted cautiously. The analysis was not used to infer separate main effects or interactions of resin type, modifier type, and UV exposure.

## 3. Results and Discussion

### 3.1. Chemical Composition (XRF)

In the present study, a detailed analysis of chemical composition was performed only for the reference concrete and selected specimens treated with the selected protection systems (Table 2). This reflects the fact that, in oxide composition analyses, the dominant components remain minerals characteristic of concrete, while the resins themselves, as organic materials, do not form significant crystalline phases that could be clearly identified using X-ray diffraction techniques. This implies that, regardless of the applied protection system, the main analytical signal originates from the concrete substrate, and any differences in chemical composition between the samples are primarily associated with its inherent heterogeneity and the local influence of the surface treatment.

All analysed samples exhibited similar oxide compositions, in which SiO_2_ was the dominant component (62.08–69.57%, depending on the sample). This is consistent with the mineralogy of Portland cement and siliceous aggregates. The second major component was CaO, with a content ranging from 10.33 to 15.44%. The lowest value was recorded for the E + ME sample (10.33%), while the highest was observed for the E sample (15.44%), which may be associated with differences in the content of portlandite and calcite phases in the analysed regions. Attention should also be given to the presence of Al_2_O_3_ (4.56–5.58%), Fe_2_O_3_ (2.54–3.16%), and K_2_O (1.37–1.84%), which are characteristic constituents of cement mineral phases (including aluminosilicates and calcium ferrites). Their contents vary within relatively narrow ranges, indicating the stability of the chemical composition of the concrete regardless of the applied coating. The remaining oxides (MgO, TiO_2_, MnO, P_2_O_5_, SO_3_) were present in trace amounts and are unlikely to play a major role in corrosion protection mechanisms. In contrast to the reference concrete, coated samples exhibited noticeably higher values of loss on ignition (LOI). This increase can be attributed to the presence of resins and organic modifiers, which introduce additional components into the analysed surface layer that undergo thermal decomposition or volatilisation during ignition. The major-oxide contents remained comparable across the samples because the dominant contribution of the concrete matrix masked the effects of components originating from the protective products. These results indicate that the applied protection systems do not significantly affect the main crystalline products of concrete. The observed variations in oxide content fall within the typical range associated with cement and aggregate composition. Consequently, the observed differences in protective performance are primarily related to modifications in coating morphology, continuity, and barrier properties, rather than to significant changes in the chemical composition of the concrete substrate itself.

### 3.2. Phase Composition (XRD)

**Figure 4:**
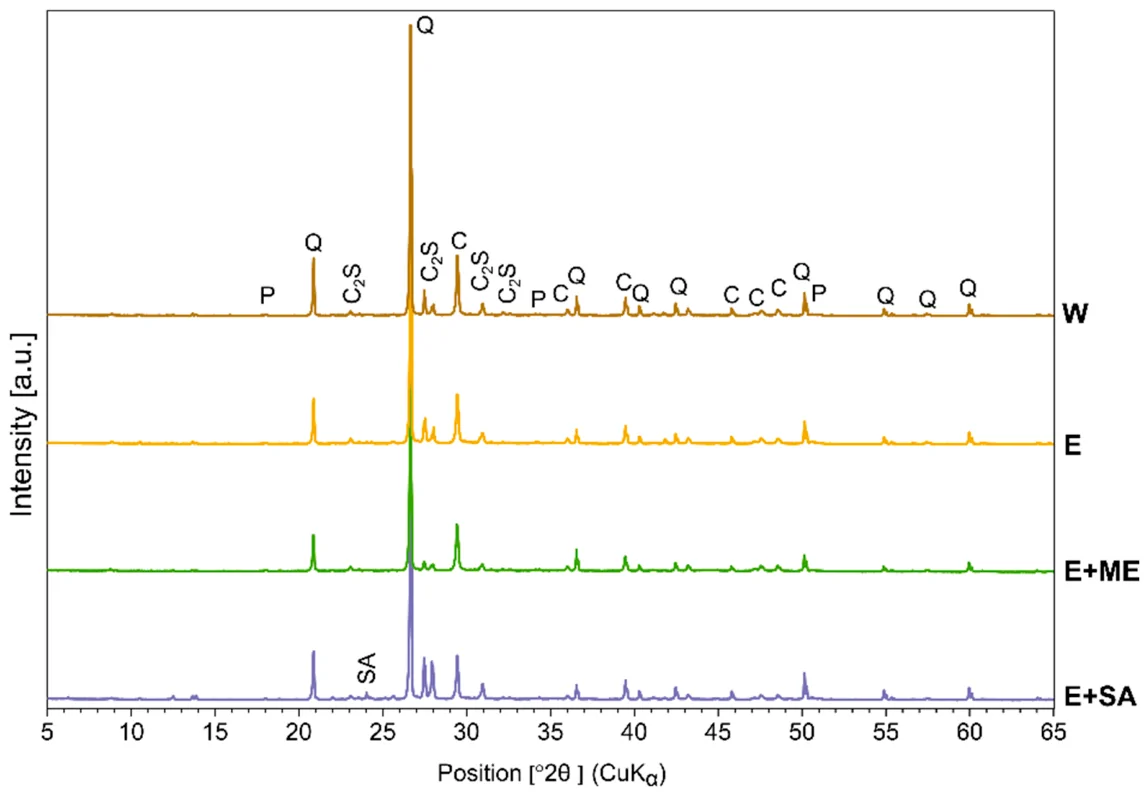
X-ray diffraction (XRD) patterns of the reference and treated concrete specimens; designations: Q—quartz, C—calcite, C_2_S—calcium silicate, P—portlandite, SA—stearic acid.

The results of the phase-composition analysis are presented in Figure 4. The XRD patterns of all analysed specimens were dominated by quartz reflections originating from the aggregate. Calcite, portlandite, and residual calcium silicate phases were also identified, and their characteristic peak positions were comparable among the investigated systems. No additional crystalline phases attributable to reactions between the protective products and the cementitious substrate were detected.

The epoxy, silane-based, silicic-acid-ester, and fatty-acid-containing systems were predominantly organic or poorly crystalline. Moreover, their contribution to the total mass of the powdered near-surface specimen was small relative to that of the concrete substrate. Consequently, these components were not expected to produce intense, well-defined diffraction peaks. The XRD results therefore indicate that the protection systems did not cause detectable changes in the principal crystalline phases of the concrete within the sensitivity of the method.

Minor differences in the relative intensities of the C_2_S, calcite, portlandite, and quartz reflections were observed. However, because no quantitative phase analysis, internal standard, or replicate scans were performed, these variations cannot be interpreted as changes in phase content. They most likely reflect the inherent local heterogeneity of concrete, including differences in the aggregate-to-cement-paste ratio, sampling depth, particle-size distribution, and preferred orientation. In particular, the C_2_S/calcite peak-intensity ratio observed for E + SA should not be attributed to inhibition of hydration, because the protective treatment was applied only after 28 days of concrete curing and subsequent drying.

Taken together with the XRF and EDS results, the XRD patterns show no systematic evidence of mineralogical alteration of the concrete substrate. Accordingly, the differences in apparent wettability, surface free energy, and morphology are interpreted as consequences of surface coverage and the physicochemical properties of the protection systems rather than changes in the crystalline products of cement hydration.

### 3.3. Apparent Static Water Contact Angle and Surface Free Energy Before and After UV Ageing

**Figure 5:**
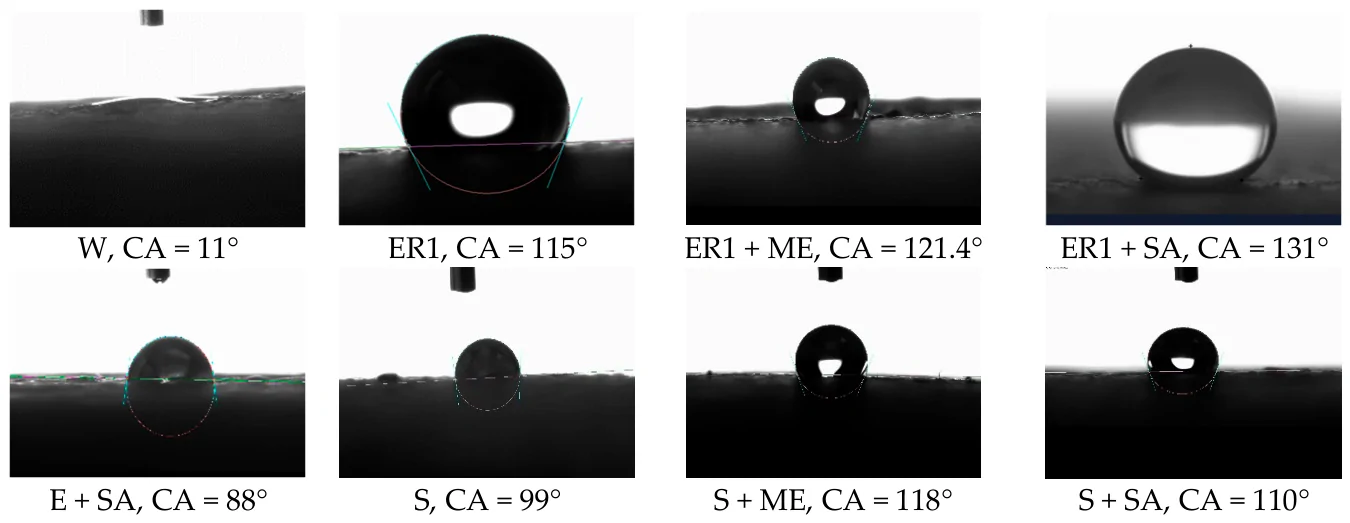
Representative water-droplet images obtained during apparent static water contact-angle measurements of the investigated concrete surface protection systems. For each system, the image corresponding to a measurement representative of the group mean was selected.

Figure 5 presents representative images from the apparent static water contact angle measurements.

**Figure 6:**
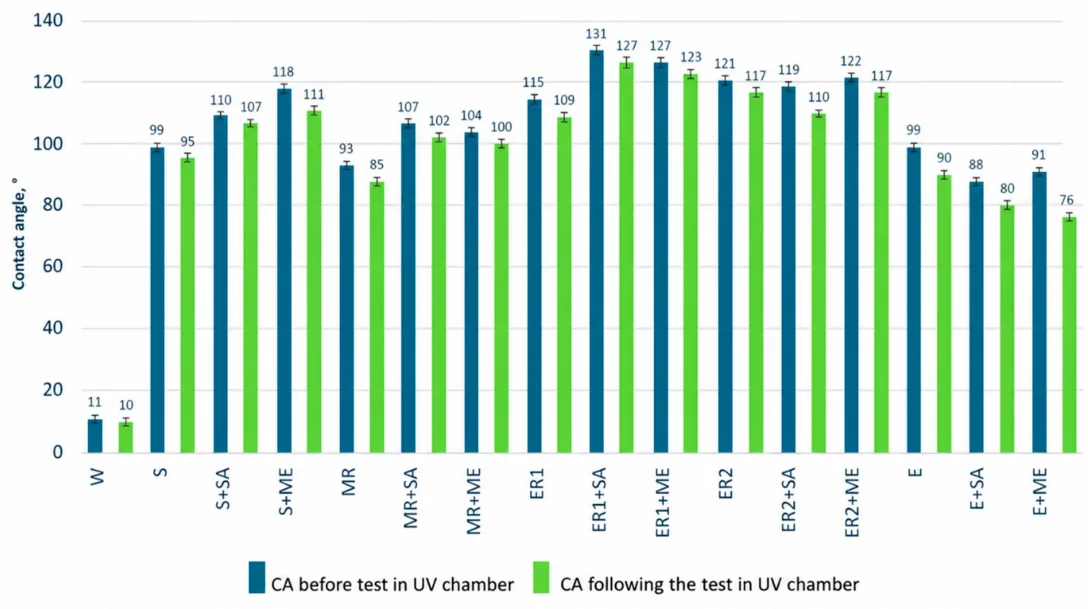
Apparent static water contact angle before and after UV ageing.

The CA results obtained before and after UV ageing were used to assess changes in coating wettability during ageing. The results are shown in Figure 6.

The reference concrete exhibited hydrophilic behaviour (CA < 90°), with an apparent water contact angle of approximately 11°. Descriptive statistics revealed marked variation in CA between the reference concrete and specimens treated with the different protection systems. All unmodified systems yielded CA values above 90°. Numerically, the methyl-ester-modified systems had CA values ranging from 91° to 127°. Among the investigated coating systems, ER1 + SA exhibited the highest observed mean contact angle of 131°. For E, the addition of stearic acid was associated with a numerical decrease in CA of approximately 12%. Concretes coated with epoxy resins exhibited the next highest average CA values (ER2: 121°, ER2 + ME: 122°, ER2 + SA: 119°). The lowest average contact angles were observed for concretes treated with silicic acid esters (E: 99°, E + ME: 91°, E + SA: 88°), as well as for concrete with MR (93°) and S (99°) resins.

**Figure 7:**
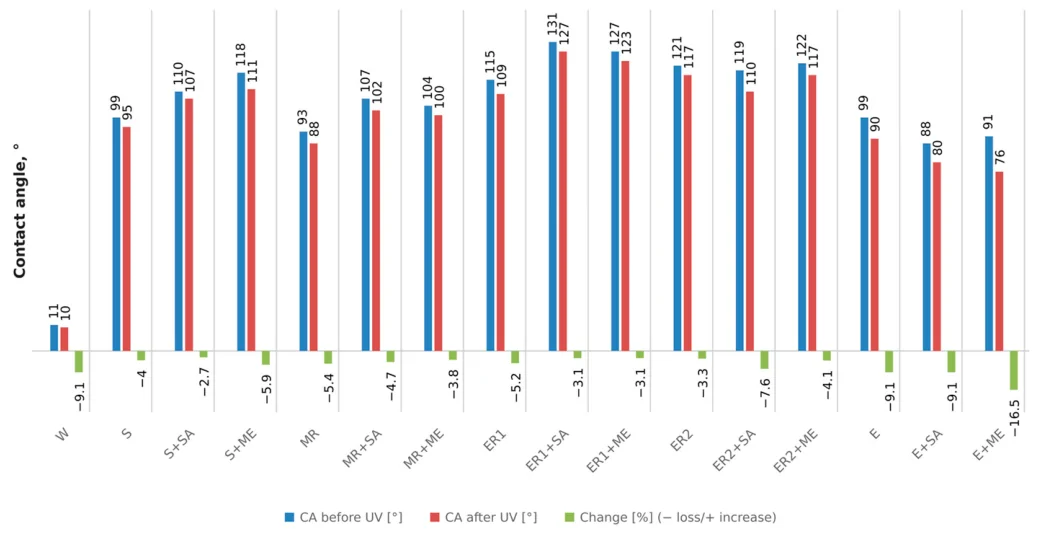
Apparent static water contact angle (CA) and percentage changes after UV ageing.

The Kruskal–Wallis test (Table 3) indicated overall differences in CA among the 16 treatment systems, H(15) = 43.175, *p* < 0.001. This result demonstrates that at least one group differed from the others but does not establish independent effects of resin type or modifier type. Dunn’s post hoc test identified significant differences only between W and ER1 + SA (p_adj_ = 0.017) and between W and ER1 + ME (p_adj_ = 0.033). No statistically significant differences were demonstrated between the modified and unmodified variants of the same resin. Descriptively, ER1 + SA exhibited the highest observed mean CA of 131°. After UV ageing, small numerical decreases in contact angle were observed for most coating systems; owing to the limited number of independent specimens, these changes are interpreted descriptively. For example, CA decreased by 16.5% for E + ME, whereas the change for S + SA was 2.7%. Overall, the results suggest that most systems retained a high proportion of their initial apparent contact angle after UV ageing. Percentage changes in CA are presented in Figure 7.

Descriptive statistics showed marked variation in apparent static water contact angle (CA) after UV ageing between the reference concrete (W) and specimens treated with the different protection systems. The reference concrete exhibited the lowest average contact angle after UV exposure (10°) and, at the same time, showed the lowest variability among all samples (SD = 2.646). The highest average CA values after UV ageing were obtained for concretes coated with epoxy resins (ER1: 109°, ER1 + ME: 123°, ER1 + SA: 127°). The lowest variability was observed for concretes with methacrylate resin (MR: SD = 1.732), although these systems also exhibited relatively low CA values (88°).

After UV ageing, the Kruskal–Wallis test (Table 4) indicated overall differences among the treatment systems, H(15) = 43.598, *p* < 0.001. Significant pairwise differences were limited to W versus ER1 + SA (p_adj_ = 0.020) and W versus ER1 + ME (p_adj_ = 0.031). This analysis compares the systems after ageing and should not be interpreted as a statistical test of the UV exposure effect. Therefore, the changes in CA recorded before and after UV ageing are discussed as descriptive within-system changes.

**Figure 8:**
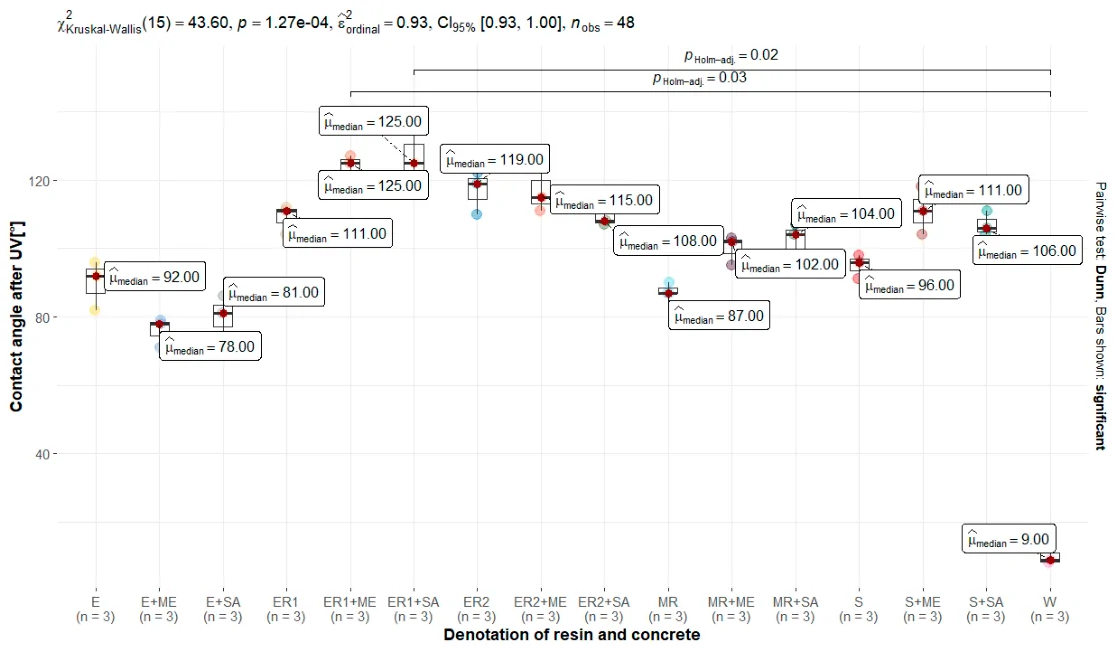
Pairwise comparison of CA values after UV ageing (Dunn’s test).

The largest differences in CA values occurred between the reference concrete (W) and the concrete coated with epoxy resin modified with stearic acid (ER1 + SA). The latter exhibited the highest average CA values, whereas the lowest values were recorded for the reference concrete (Figure 8). This confirms the statistical significance of the differences between these two groups (adjusted *p* = 0.020). Significant differences were also observed between the concrete coated with epoxy resin modified with methyl esters (ER1 + ME) and the reference concrete (W) (adjusted *p* = 0.031). The remaining comparisons were not statistically significant (adjusted *p* > 0.05).

It should be emphasised that the measured values represent apparent static water contact angles rather than equilibrium thermodynamic contact angles. Concrete surfaces are rough, porous, and chemically heterogeneous, and the measured angle may therefore be affected by local surface morphology, contact-line pinning, capillary absorption, and spreading. The use of a small droplet volume and a fixed evaluation time reduced, but did not eliminate, these effects. Accordingly, the results are primarily suitable for comparative assessment of the investigated protection systems tested under identical conditions. Dynamic measurements, including advancing and receding angles, contact-angle hysteresis, and the evolution of contact angle and droplet volume over time, would provide a more comprehensive description of the wetting behaviour and should be considered in future studies.

**Figure 9:**
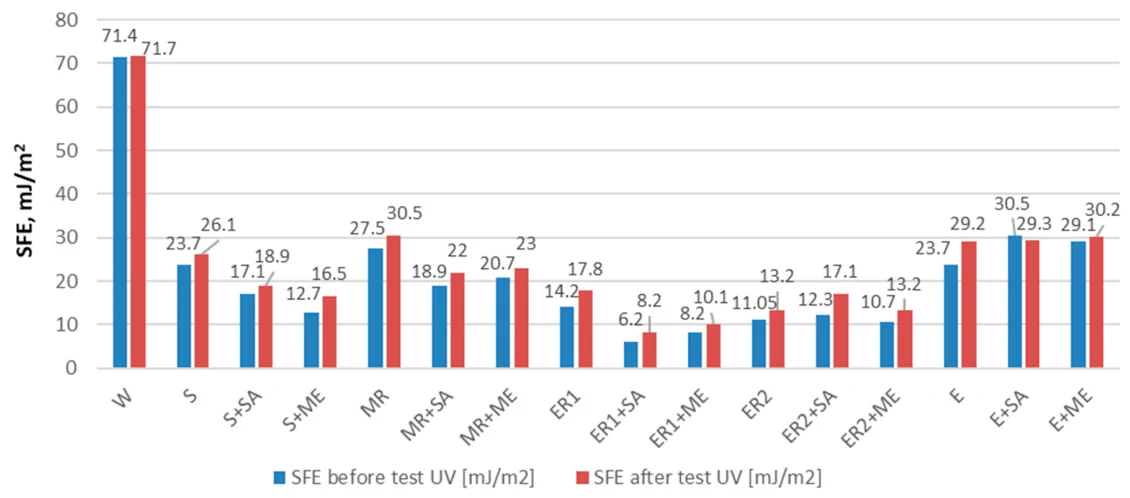
SFE values of concrete calculated using the Neumann method.

Figure 9 presents the values of the surface free energy SFE calculated using the Neumann method based on the formula:

$$\cos\theta_{w}=\left[e^{-0.000125{\left(\gamma_{s}-\gamma_{w}\right)}^{2}}2\sqrt{\frac{\gamma_{s}}{\gamma_{w}}}-1\right]$$

where $\gamma_{s}$—total SFE value, $\gamma_{w}$—SFE of distilled water (72.8 mJ/m^2^), θ_w_—water wetting angle.

The untreated concrete had the highest total surface free energy (SFE), at 71.4 mJ/m^2^ before testing in the UV ageing chamber. As the contact angle increased, the SFE values decreased. The modified epoxy systems had the lowest SFE values (ER1 + ME: 8.2 mJ/m^2^; ER1 + SA: 6.2 mJ/m^2^). Low water adhesion was also observed for the ER2 resin. Across the investigated systems, higher surface roughness was associated with greater apparent wettability and higher SFE. A slight increase in SFE was also observed after UV ageing, consistent with a small reduction in surface hydrophobicity.

The results were compared with previous studies to place them in a broader scientific context and identify potential research gaps. Yang et al. [35] showed that the incorporation of stearic acid-modified mica into cement mortars reduced water absorption, improved hydrophobicity, and enhanced frost resistance under saline freeze–thaw conditions. SEM observations also indicated a denser C-S-H structure and reduced crack development in modified mortars.

The hydrophobic effect of stearic acid has also been confirmed for other mineral and cementitious systems. Research conducted by Hu et al. [36] demonstrated that calcium carbonate coated with stearic acid achieved a water CA of 143°, while Liu et al. [37] demonstrated superhydrophobic (CA = 158°) and self-cleaning properties of stearic acid-modified coatings. Similar improvements, including reduced water absorption and increased contact angle, were also observed in stearic acid-modified mortars by Feng et al. [38]. In hybrid nanoparticle coatings, Li et al. [39] confirmed that stearic acid modification strongly enhanced surface hydrophobicity (CA = 160°). Comparable effects were reported for stearic acid-based coatings on steel by Zhang et al. [40] and for stearic acid-treated concrete surfaces in another study [41]. Overall, these findings confirm that stearic acid is an effective hydrophobic modifier; however, its efficiency depends on the type of substrate, modification method, dosage, and exposure conditions.

### 3.4. Resistance to Dirt (Ink and Coal Dust)

**Figure 10:**
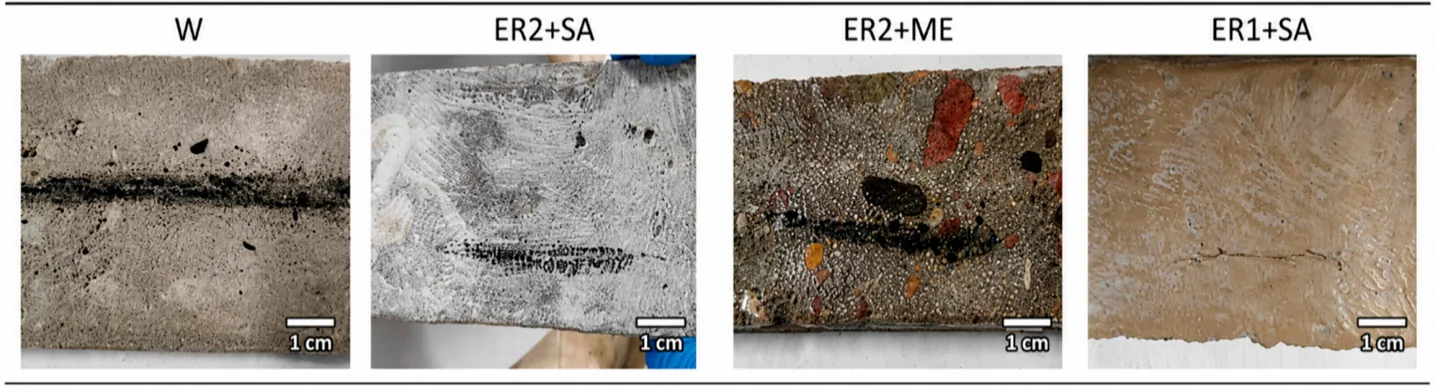
Representative specimens during coal-dust resistance testing.

Representative images from the tests are presented in Figure 10. In the coal-dust test, W concrete was permanently stained (Figure 10), as were specimens treated with systems exhibiting higher surface roughness, such as ER2 + SA and ER2 + ME. The ER1-based systems showed the greatest resistance to coal dust, both before and after modification with fatty acids. In these cases, dust did not adhere to the coating surface and could be easily removed.

**Figure 11:**
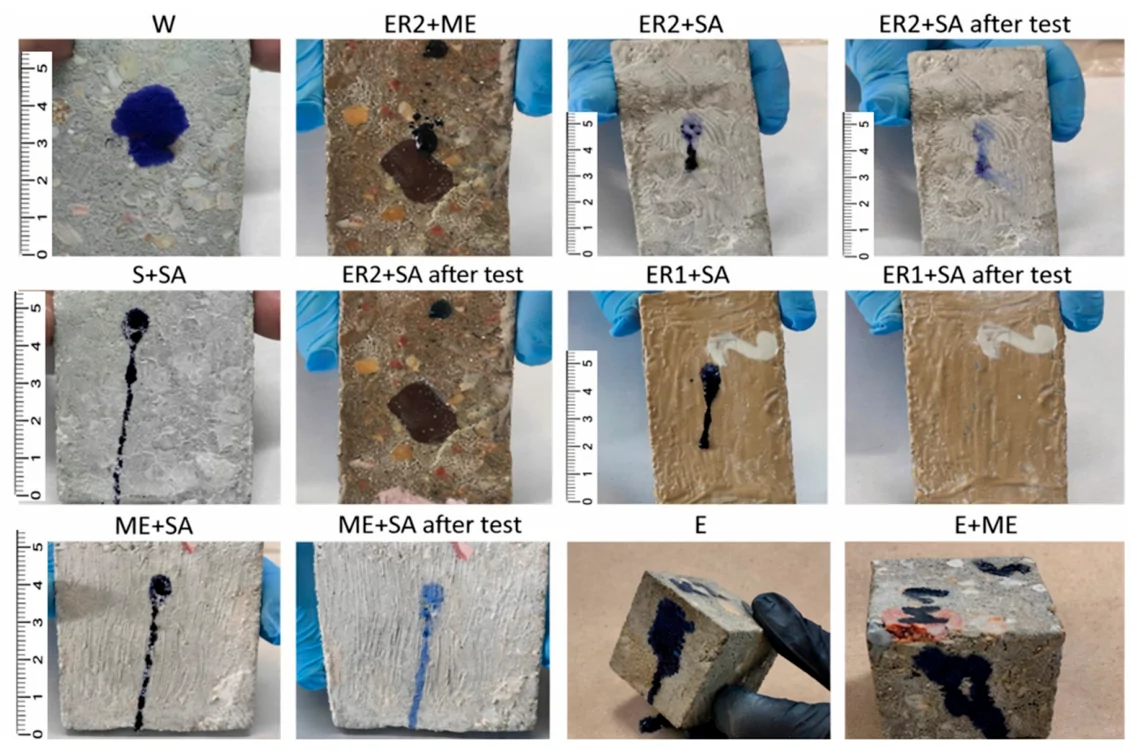
Representative specimens during ink-staining resistance testing.

In contrast, ink caused permanent staining of the concrete surface (Figure 11), and it was not possible to remove it using a cloth.

Resin systems based on silanes (Ss) and silicic-acid-ester-based (E) systems also failed to protect the surface effectively, even after modification with fatty-acid-derived additives. The MR + SA resin exhibited hydrophobic behaviour, with ink flowing off the surface; however, complete removal of contamination could not be removed completely. By contrast, ER1 + SA, ER1 + ME, and ER2 + ME showed excellent stain resistance—the ink ran off the surface, and any remaining traces were readily removed. Resistance to staining results from several factors. One of the key parameters is surface roughness. Coal dust and ink can become retained within surface irregularities, while the concrete is covered to different degrees by the protective product. In this respect, the S and E series were less effective at preventing fouling. This may be related to their low viscosity, which allows deeper penetration into the concrete structure. As a result, although these systems effectively limit water absorption, they may lead to the formation of a relatively rough underlying surface texture remains comparatively pronounced and can promote contaminant retention through mechanical adhesion. The addition of stearic acid (SA) and methyl esters (MEs) slightly improved this behaviour, possibly because their hydrocarbon chains lowered the surface affinity for water and contaminants.

Organic resin-based systems generally provide better resistance to contamination. In the present study, this trend was observed for the MR series, which formed a smoother coating on the concrete surface. These systems did not penetrate as deeply into surface irregularities, and their long molecular chains reduced the tendency for dirt adhesion. Another important factor affecting fouling resistance is the intrinsic texture of the resin. The more vitreous (glassy) the surface, the easier it is to remove contaminants. In this respect, epoxy resins generally perform best, as the cross-linking process results in a smooth, glassy surface to which contaminants do not adhere or from which they readily flow off. In the present study, the addition of SA and ME further enhanced this effect, and the ER1 and ER2 series exhibited the highest resistance to fouling.

According to Kong et al. [40], the hydrophobicity of concrete modified with stearic acid (SA) can be attributed to two main factors. First, the high contact angle (CA) was influenced by changes in surface roughness. An increase in micro- and nanoscale roughness increases the contact angle on the surface, thereby enhancing hydrophobicity. Second, SA reacts with the C-S-H gel, modifying its chemical structure by introducing hydrophobic functional groups such as -CH_3_ and -CH_2_, which have lower surface energy than hydroxyl groups (-OH). Consequently, SA reduces the surface energy of concrete, enhancing its hydrophobic properties. XRD analysis further showed that the calcium aluminosilicate hydrate (C-A-S-H) gel is surrounded by a hydrophobic layer, indicating a high degree of water repellency. The results also confirmed the successful formation of a superhydrophobic coating using SA. Feng et al. [42] reported that zinc-modified carbon steel treated with stearic acid exhibited a near-surface structure rich in entrapped air within its pores. This feature limited the effective contact area between water and the substrate, thereby enhancing the superhydrophobic performance of the nanocoating. The hydrophobic properties of surfaces modified with stearic acid are primarily attributed to their surface chemistry, where long alkyl chains lower the surface energy [43]. Furthermore, interactions between the carbonyl groups of stearic acid and hydroxyl groups present in organosilicon compounds contribute additionally to this effect [44].

Kong et al. [41] attributed the high contact angle of their stearic-acid-modified coating to the combined effects of low surface energy and a deliberately developed micro-/nanostructure. Under such conditions, hierarchical roughness may facilitate air entrapment beneath the water droplet and promote a Cassie–Baxter-type wetting state. This mechanism should not be directly equated with the roughness measured in the present study. Here, the optical roughness parameters primarily describe micrometre-scale pores, valleys, and coating discontinuities, which may promote contact-line pinning and local water penetration rather than stable air retention. Therefore, the results of Kong et al. are cited as an example of a different, engineered wetting regime and not as a direct explanation of the inverse Ra–CA relationship observed for the present coating systems. The enhanced antifouling performance was attributed to the water-repellent nature of the coating and its low interfacial adhesion. Other researchers have used methyl esters derived from vegetable oils as modifiers of commercial water repellents [4]. In these studies, concrete water absorption was approximately halved. Systems modified with methyl esters exhibited higher watertightness and up to 72% greater frost resistance compared with reference concrete. Wang et al. [45] demonstrated that coatings modified with fatty acids exhibit excellent self-cleaning performance, with contact angles reaching up to 164° and corrosion resistance of up to 99.98% in a NaCl solution.

### 3.5. Surface Roughness (Optical Microscopy)

**Figure 12:**
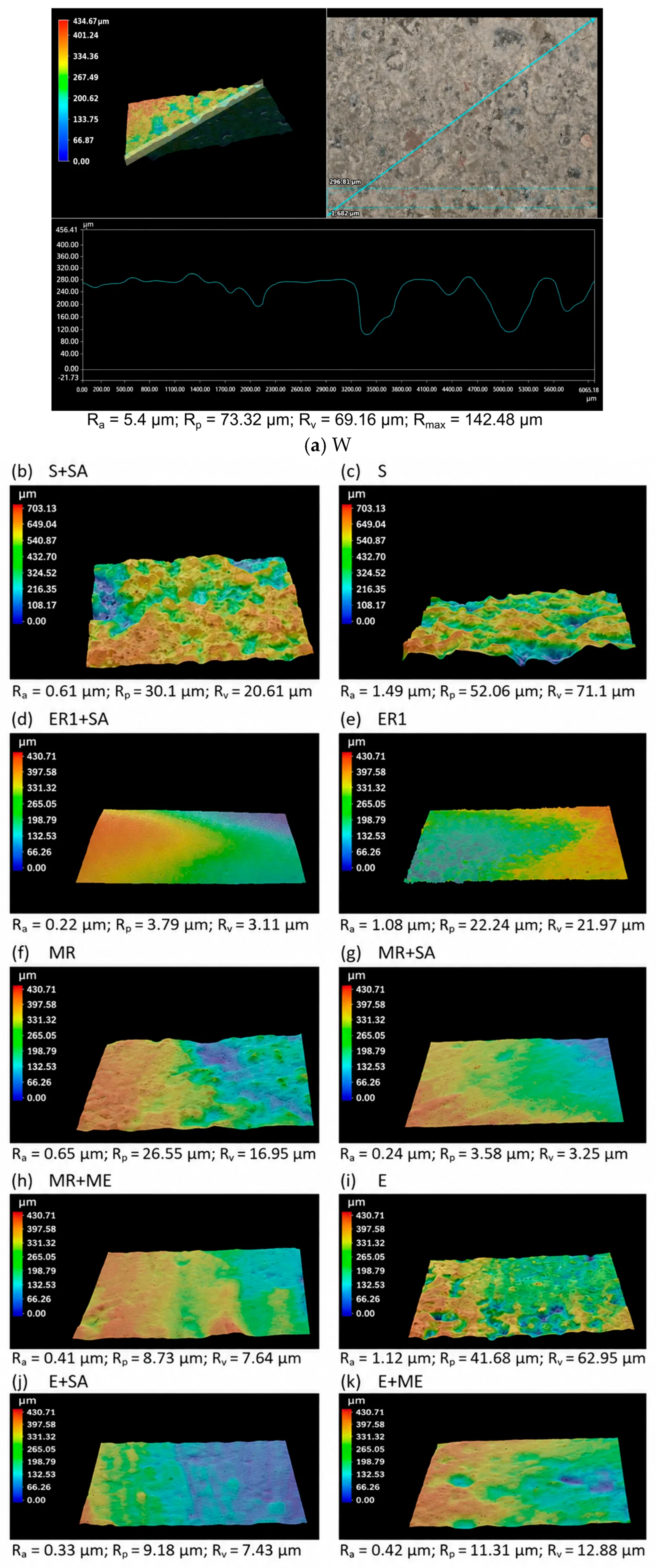
Surface roughness of resin-based coatings on concrete (3D).

Figure 12 presents a comparison of the surface morphology of the reference sample (W) and selected protective coatings. The obtained roughness results clearly indicate differences associated with coating type and chemical modification. The reference concrete was characterised by the highest values of roughness parameters, i.e., R_a_ = 5.4 µm, R_p_ = 73.32 µm, and R_v_ = 69.16 µm (Figure 12a). Such high values reflect the irregular structure, typical of porous material such as concrete. This type of morphology may promote moisture retention and the penetration of aggressive agents, making the material susceptible to degradation and therefore requiring protection. The application of silane-based coatings (S) resulted in a noticeable smoothing of the surface; however, the structure remained clearly porous (R_a_ = 1.49 µm). The silanes did not block the pores, indicating that their primary function was hydrophobisation rather than pore sealing (Figure 12b,c). Only the modification of silanes with stearic acid (S + SA) led to a significant reduction in roughness parameters to R_a_ = 0.61 µm, R_p_ = 30.1 µm, and R_v_ = 20.61 µm, suggesting partial filling of surface irregularities and more uniform coverage.

A similar trend was observed for epoxy coatings (ER1). The epoxy resin itself reduced surface roughness compared with the reference concrete; however, only its modification with stearic acid (ER1 + SA) resulted in the most uniform and smoothest surface (R_a_ = 0.22 µm), corresponding to a more than 24-fold reduction relative to the reference concrete (Figure 12d,e). Such low roughness indicates a more uniform surface with fewer visible discontinuities capable of retaining water and contaminants. Together with the direct water-transport results reported in Reference [31], this morphology supports the favourable moisture-protection potential of the coating. Direct initiation or propagation of reinforcement corrosion was not evaluated. Methacrylate coatings (MRs) also exhibited a beneficial effect of modification. The introduction of stearic acid reduced the average roughness from R_a_ = 0.65 µm to R_a_ = 0.24 µm (a 63% decrease), while methyl esters reduced it to R_a_ = 0.41 µm (a 37% reduction). Importantly, the maximum peak height decreased from R_p_ = 26.55 µm to R_p_ = 3.58 µm, confirming a substantial improvement in surface smoothness and tightness (Figure 12f–h).

In the case of silicic acid esters (E), the unmodified coating initially exhibited relatively high roughness (R_a_ = 1.12 µm). However, modification with stearic acid (E + SA) resulted in a reduction in average roughness by as much as 70% (R_a_ = 0.33 µm), indicating a combined smoothing effect of the ester and stearic acid in sealing deeper microscopic irregularities. Methyl esters reduced roughness by 62% (R_a_ = 0.42 µm) (Figure 12i–k). For coated concrete surfaces, the R_max_ parameter is more relevant than the average roughness R_a_ because it captures extreme peak-to-valley variation. R_a_ is an averaged parameter and may not reflect isolated deep valleys. A high R_max_ value may therefore indicate localised surface defects that can retain water or provide transport pathways. Even when the average roughness (R_a_) is low, a high R_max_ value may indicate the presence of deep, localised defects that can act as pathways for moisture, salts, and aggressive agents. Impregnation systems, particularly high-molecular-weight resins, often exhibit a limited ability to penetrate deep, narrow pores; consequently, high R_max_ values), which may lead to localised regions of reduced coverage. The reference sample (W) exhibited the highest R_max_ value (142.48 µm), indicating a highly developed surface topography with numerous deep irregularities, typical of unprotected concrete. The ER1 resin effectively smoothed the surface, reducing R_max_ to 44.21 µm. The addition of stearic acid in the ER1 + SA sample further reduced this parameter to 6.9 µm, indicating a smoother surface with fewer extreme irregularities.

In summary, fatty-acid modification, particularly with stearic acid, was descriptively associated with improved surface topography across the analysed coating systems. The results indicate that:

The reference concrete had the highest roughness values, consistent with an irregular surface capable of retaining moisture and contaminants.

The organosilicon systems (silane and silicic acid ester) reduced surface roughness but did not produce complete surface sealing.

The epoxy and methacrylate coatings modified with fatty-acid-derived additives produced the most compact and smoothest surfaces.

The lowest R_a_ value was obtained for the ER1 + SA coating (R_a_ = 0.22 µm), corresponding to the smoothest measured surface.

Reduced surface roughness may limit the retention of water, contaminants, and microorganisms; however, it does not independently demonstrate a lower risk of reinforcement corrosion.

The results demonstrate that chemical modification influenced the wettability, roughness, morphology, and visible service condition of the investigated surfaces. Complementary transport and durability tests reported in Reference [31] confirmed reduced water uptake and improved resistance to freeze–thaw cycles and salt crystallisation for selected systems. Long-term coating adhesion and electrochemical reinforcement corrosion were outside the scope of the present study.

**Figure 13:**
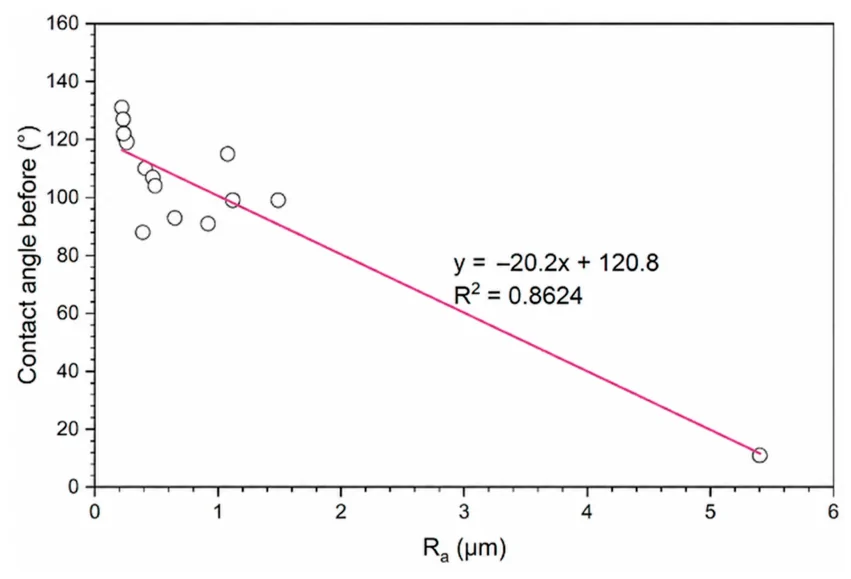
Correlation between the apparent static water contact angle (CA) and average surface roughness (R_a_).

Figure 13 shows an inverse empirical relationship between the apparent static water contact angle and the average profile roughness (R_a_) across the investigated protection systems (R^2^ = 0.8624). However, the regression combines surfaces that differ simultaneously in chemical composition, coating type, modifier, and degree of substrate coverage. It therefore does not demonstrate an independent causal effect of roughness on wettability. Within the present dataset, lower (R_a_) was generally associated with more uniform coverage by low-surface-energy coating components, whereas higher (R_a_) reflected greater exposure of the porous cementitious substrate and more pronounced surface discontinuities.

The linear relationship is characterised by a high coefficient of determination (R^2^ = 0.8624) and is described by the equation *y* = –20.2*x* + 120.8. The reference concrete exhibited the highest average roughness (R_a_) and the lowest water contact angle (11°). In contrast, the lowest R_a_ values were obtained for the ER1 + SA coating (R_a_ = 0.22 µm), which was characterised by the highest contact angle (CA = 131°).

The relationship between surface roughness and wettability depends on both the intrinsic surface chemistry and the wetting regime. In the Wenzel model, complete contact occurs between the liquid and the surface topography, and roughness amplifies the intrinsic wetting behaviour of the material. In contrast, the Cassie–Baxter model assumes a composite solid–liquid–air interface, in which air retained within an appropriate hierarchical texture can substantially increase the apparent contact angle.

The present results do not provide sufficient evidence to assign the investigated surfaces to a Cassie–Baxter state. The measured values represent apparent static water contact angles, and neither contact-angle hysteresis, sliding angle, nor the presence of a stable air layer beneath the droplets was determined. Moreover, the R_a_ values obtained by optical profilometry describe micrometre-scale irregularities, including pores, valleys, exposed aggregate grains, and coating discontinuities, rather than a deliberately engineered micro-/nanostructure.

Within the investigated coating systems, an increase in R_a_ was associated with lower apparent contact angles and higher apparent surface free energy. This trend can be explained by greater exposure of the hydrophilic cementitious substrate, increased contact-line pinning, and possible local water penetration into surface irregularities. Conversely, lower R_a_ generally indicated more uniform coverage of the substrate by a low-surface-energy organic phase. Because the investigated surfaces differed simultaneously in chemical composition, coating continuity, and topography, the observed relationship should be interpreted as an empirical association rather than as the independent effect of roughness alone.

**Figure 14:**
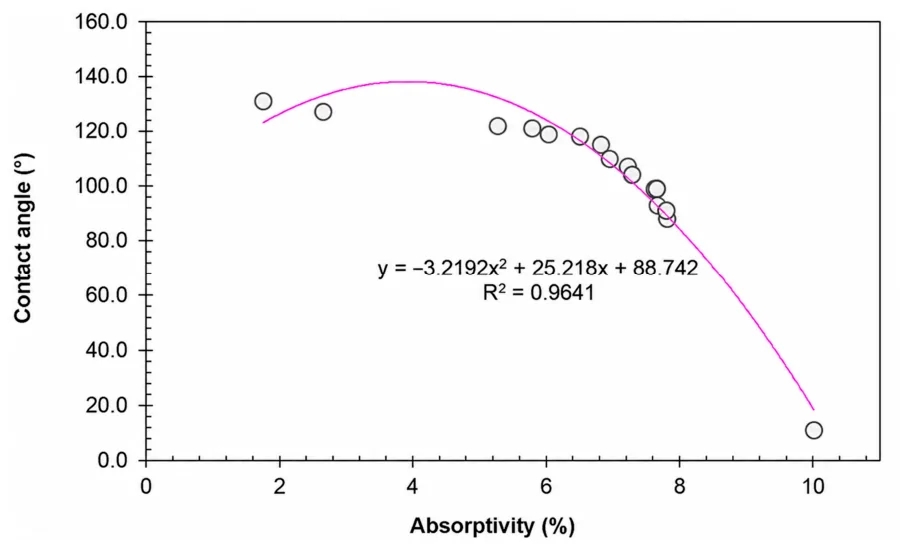
Secondary correlation between the apparent static water contact angle measured in the present study and water absorptivity previously reported for the same protection systems in Reference [31]. The regression analysis was performed in the present study.

To evaluate the association between surface wettability and macroscopic water uptake, the apparent static water contact angles measured in the present study were correlated with the water absorptivity values previously reported for the corresponding protection systems in Reference [31]. Thus, the absorptivity results do not constitute a new dataset generated in the present study but were used in a new secondary correlation analysis. This relationship is expressed by the polynomial function *y* = −3.2192*x*^2^ + 25.218*x* + 88.742, with a very high coefficient of determination (R^2^ = 0.964) (Figure 14).

### 3.6. Microstructural Studies (SEM)

**Figure 15:**
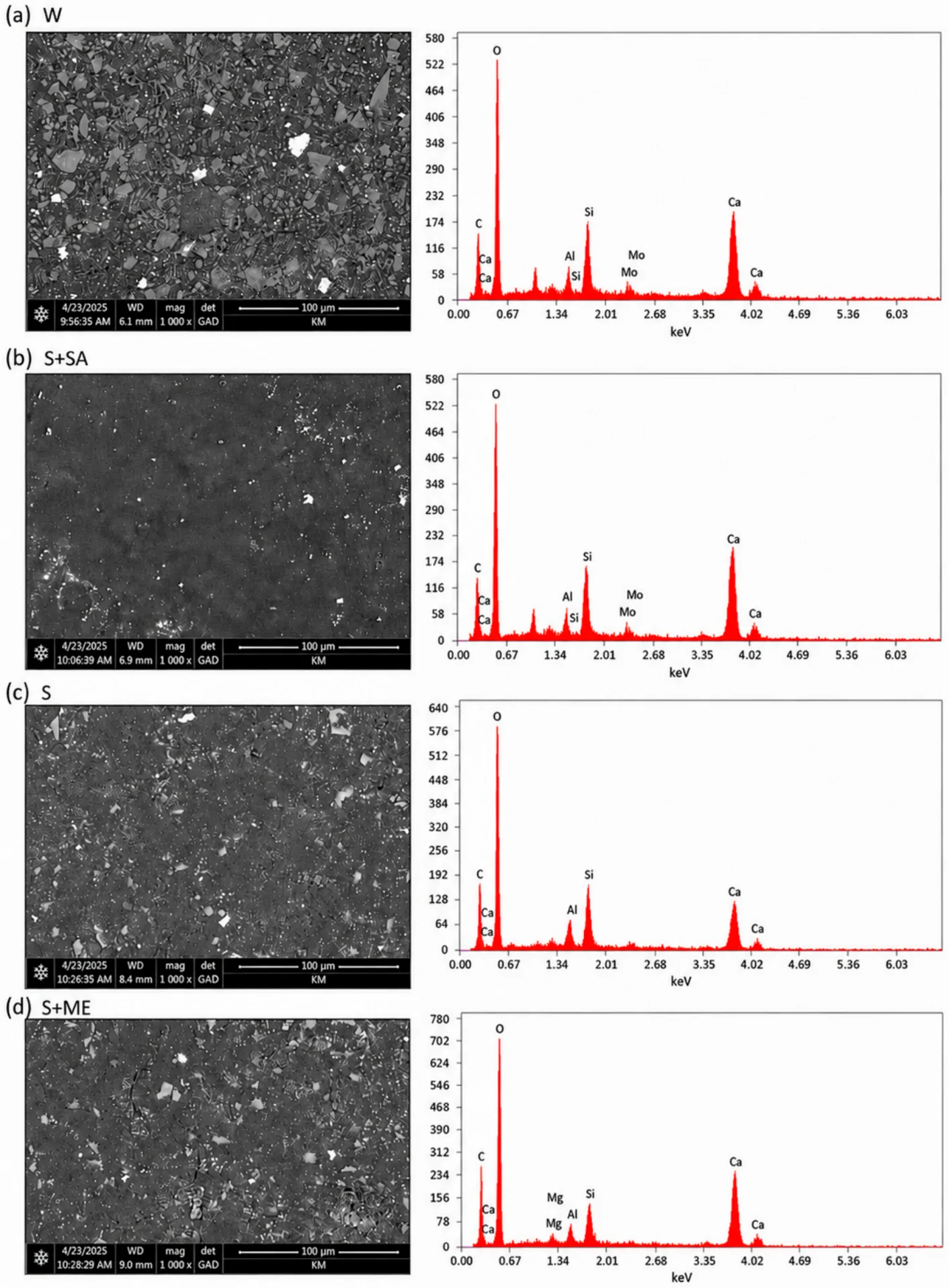

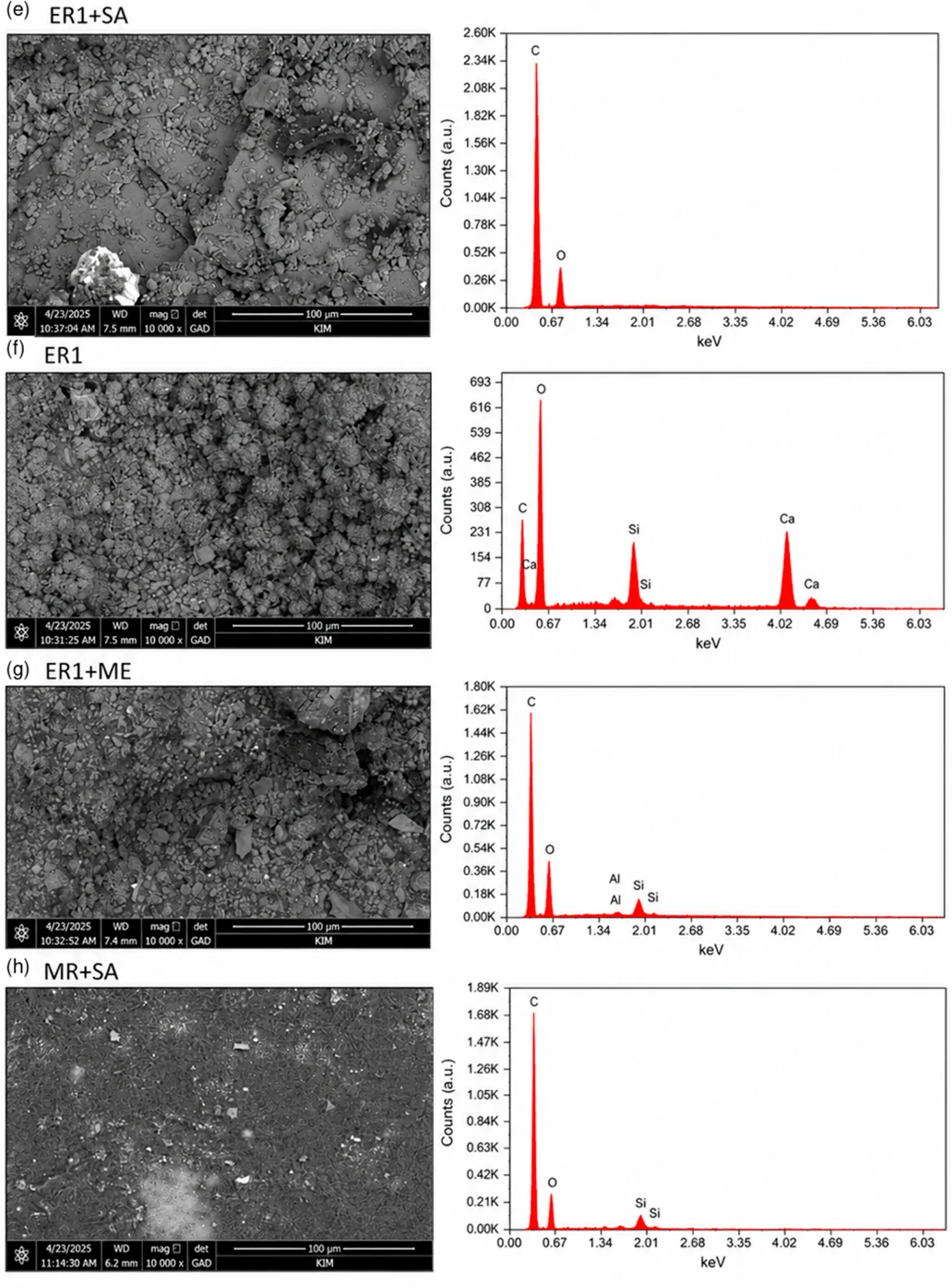
SEM micrographs and EDS analysis of the elemental composition of resin-based coatings on concrete surfaces.

Scanning electron microscopy (SEM) was used to investigate the microstructure of the coatings. Figure 15 presents micrographs of anticorrosion coatings on concrete surface at 1000× magnification.

The scanning microscopy images (Figure 15) showed differences in surface coverage and domain size among the investigated systems. The formed gel particles differ in particle size.

Plan-view SEM observations revealed substantial differences in the surface morphology of the investigated protection systems. The epoxy-treated surfaces exhibited polymer-rich morphological domains with lateral dimensions ranging from approximately 8 to 40 μm. These dimensions describe features visible in the surface plane and do not represent coating thickness or penetration depth. ER1 exhibited a granular and heterogeneous surface morphology, with local agglomerate-like regions, depressions, and visible discontinuities up to approximately 2.9 μm in width. The term “relatively uniform distribution” refers only to the general surface coverage observed within the analysed fields of view and should not be interpreted as a quantitative measure of three-dimensional coating homogeneity.

The ER1 coating exhibited a granular and clearly heterogeneous structure, with visible agglomerates, local inhomogeneities, and micro-areas of increased porosity. The ER1 epoxy-rich surface domains (Figure 15e–g) did not form a visually continuous surface layer within the examined fields of view, and gaps between particles with widths of up to 2.9 µm were observed.

The plan-view SEM observations revealed substantial differences in the surface morphology produced by the impregnation and film-forming systems. In the specimens treated with the low-viscosity silane-based and silicic-acid-ester products, aggregate grains and features of the underlying cementitious substrate remained visible. This morphology is consistent with the intended impregnation mechanism of these products, which primarily modify the walls of the near-surface capillary pores rather than forming a macroscopically thick polymer film. These plan-view observations do not quantify penetration depth or coating thickness. The favourable resistance of the corresponding systems to water uptake and penetration was independently confirmed by the direct transport measurements reported in Reference [31]. However, neither the present results nor Reference [31] provide direct electrochemical evidence of reinforcement corrosion protection.

In contrast, the epoxy systems produced polymer-rich surface domains that partly masked the original mineral topography of the substrate. Considering their film-forming character and substantially higher viscosity, particularly for ER1 (10,160 mPa·s), the observed morphology is consistent with the formation of a predominantly surface-located polymer barrier. Limited penetration or filling of larger surface pores cannot be excluded; however, penetration depth and coating thickness were not quantified in the present study.

Modification with stearic acid resulted in more uniform surface coverage and a reduction in the visible surface irregularities, particularly for ER1 + SA. Accordingly, the term “surface-sealing effect” used here refers only to the reduction in visible surface discontinuities and roughness within the examined surface area and does not imply complete closure of the internal pore network. The addition of methyl esters (MEs) did not produce marked changes in coating morphology at the micrometre scale. SEM micrographs obtained before and after modification showed comparable compactness and phase distribution, indicating good compatibility of the additive with both the epoxy matrix (ER1) and organosilicon systems (S), as well as the absence of significant phase separation. These observations suggest that MEs act primarily as modifiers of physicochemical properties rather than as agents altering at the morphological structure of the coating. No microscale agglomeration or phase separation attributable to ME was observed within the analysed surface areas. This observation suggests a relatively uniform physical distribution of the modifier but does not provide information on possible molecular-level interactions with the resin matrix. Instead, compared with solid SA, liquid ME can be dispersed more readily during sonication and can reduce resin viscosity. In contrast, stearic acid (SA) may partially crystallise, segregate, and migrate towards the surface. Methyl esters also contributed to sealing of the concrete structure, although to a lesser extent than SA. The differences in coating structure arise from the chemical nature of both modifiers of stearic acid (SA), due to their long hydrocarbon chains that may lower the surface affinity for water; however, their effects depend on resin chemistry, modifier distribution, and polar -COOH group, interact strongly with the epoxy matrix and impart pronounced hydrophobic properties to the coating. The stronger smoothing observed for SA in some systems should therefore be regarded as an empirical result rather than evidence of a specific molecular to form a stable sealing network, result in a less pronounced smoothing effect compared to STA (Figure 15b,h).

EDS analysis showed that the spectra obtained from the reference concrete and the specimens treated with the silane-based product S were dominated by elements characteristic of the cementitious substrate, primarily Si, Ca, and Al. This indicates that the mineral substrate remained within the EDS interaction volume, which is consistent with the low-viscosity impregnation mechanism and the absence of a macroscopically thick surface film.

In the epoxy- and methacrylate-treated specimens, the signals originating from the mineral substrate were substantially attenuated or locally absent. This observation is consistent with the presence of an organic, surface-dominated phase that limited signal acquisition from the underlying concrete. However, because the investigated organic coatings contain no unique elemental marker and EDS has limited sensitivity and specificity for light elements, the spectra cannot be used to determine coating thickness, penetration depth, interfacial bonding, or complete layer continuity. The EDS results are therefore interpreted only as qualitative evidence of differences in the surface regions analysed for the impregnation and film-forming systems.

The apparent static water contact angle, surface free energy, roughness, and surface morphology investigated in the present study provide information on surface wettability, coating coverage, and visible surface discontinuities. These parameters should not be interpreted as independent direct measurements of water permeability, coating adhesion, or reinforcement corrosion. However, reduced water absorptivity, water permeability, and watertightness of the corresponding protection systems were directly demonstrated in the preceding complementary study [31]. The present surface-scale results are therefore discussed together with, but clearly distinguished from, the previously published macroscopic water-transport and durability results. Quantitative information on surface topography was obtained independently using optical roughness measurements. The parameters (R_a_), (R_p_), (R_v_), and (R_max_) were used to characterise average and extreme surface irregularities. These results complement the qualitative plan-view SEM observations but do not quantify internal coating porosity, coating thickness, or penetration depth. The association between surface topography and wettability is therefore discussed as a combined effect of coating chemistry, surface coverage, and morphology rather than as a direct causal relationship between an SEM-derived homogeneity index and protective performance.

The SEM examination was limited to plan-view observations of selected surface areas. Consequently, it provides qualitative information on surface morphology, visible coverage, and local surface discontinuities but does not quantify coating thickness, penetration depth, connected porosity, or coating–substrate adhesion. Conventional EDS mapping would additionally have limited specificity because the organic coatings contain no unique elemental marker that would allow them to be distinguished unequivocally from the cementitious substrate. Dedicated cross-sectional imaging and an appropriate phase-identification or labelling procedure would be required for quantitative assessment of these parameters.

### 3.7. In Situ Assessment

**Figure 16:**
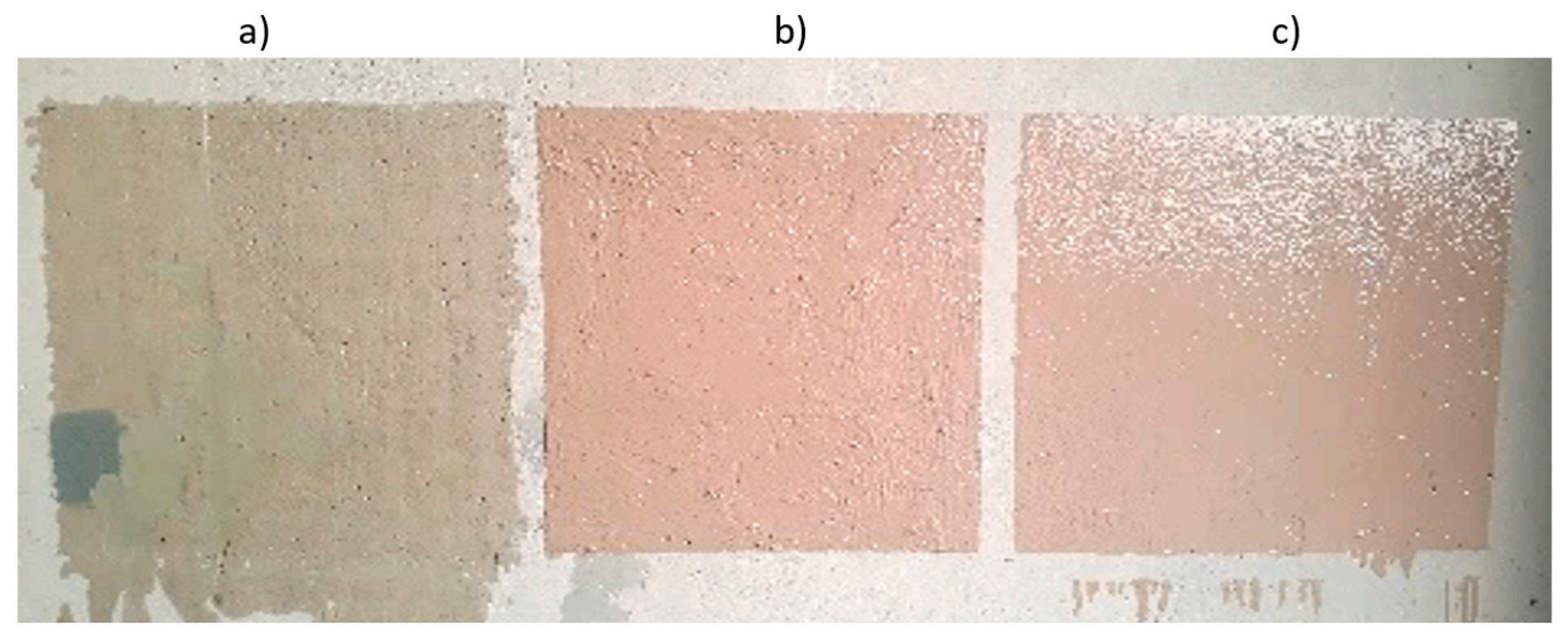
Reinforced concrete cooling-tower wall coated with modified coating systems (2024): (**a**) ER2 + ME, (**b**) ER1 + SA, (**c**) ER1 + ME.

Sections of the internal walls of the cooling tower at PGE Energia Ciepła S.A. in Lublin were coated with three modified resins: ER2 + ME, ER1 + SA, and ER1 + ME (Figure 16). The coatings were applied in two layers using a paint roller.

**Figure 17:**
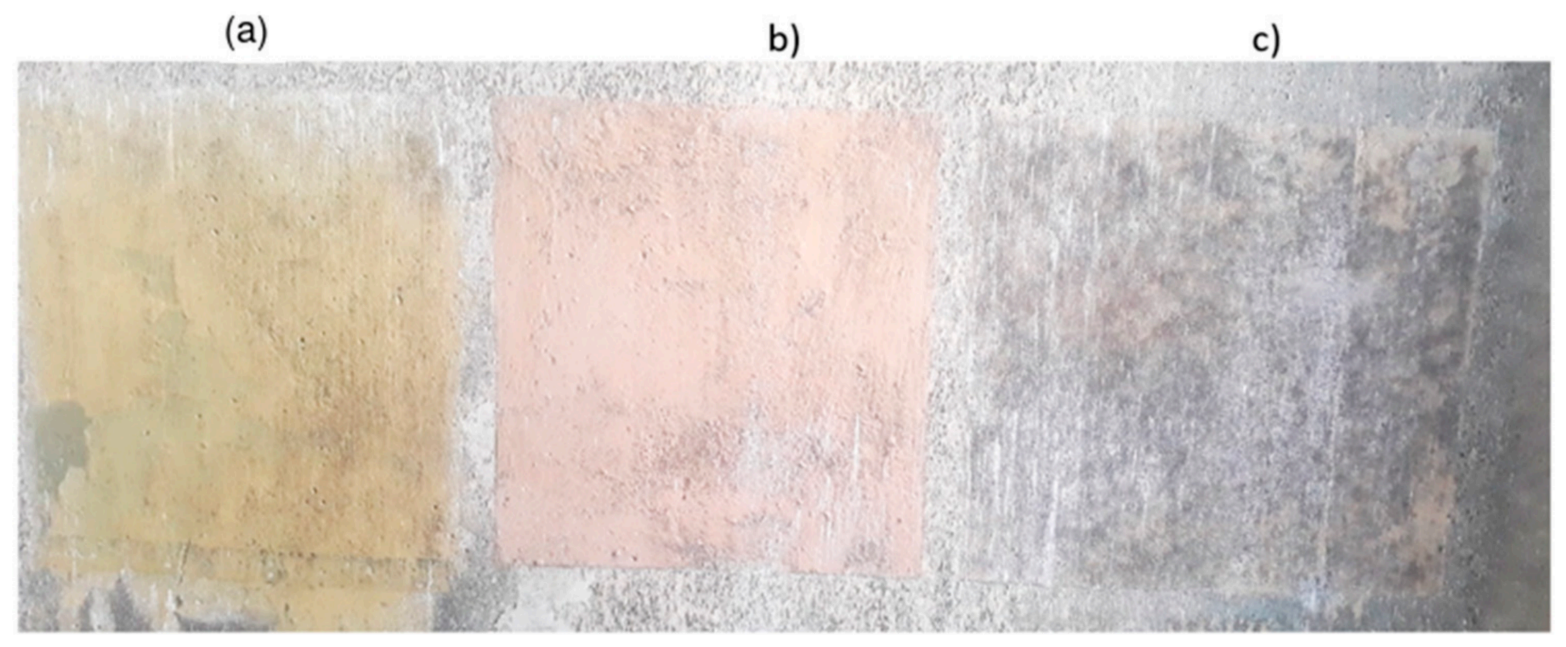
Reinforced concrete cooling-tower wall after one year of operation (2025): (**a**) ER2 + ME, (**b**) ER1 + SA, (**c**) ER1 + ME.

After the coatings had fully cured, their initial condition was assessed, and the cooling tower was returned to normal operation. After one year, the condition of the coatings on the internal cooling-tower walls was evaluated in situ. The results are presented in Figure 17.

A detailed description of the condition of the coatings after one year of operation is presented below:

- ER2 + ME coating—very good condition; best-preserved system.
- Surface appearance: sporadic, thin deposits and isolated stains; the coating remains uniform and retains a colour similar to its initial state.
- Biocorrosion: minimal colonisation; a thin biofilm that can be easily removed mechanically.
- Adhesion/integrity: good; no visible delamination; coating structure and continuity preserved.
- Possible interpretation: reduced wettability and limited adhesion of contaminants.
- ER1 + SA coating—good to moderate condition.
- Surface appearance: dispersed stains and streaks of algae in areas of water runoff; localised matting observed.
- Biocorrosion: thin biofilms present, mainly in shaded areas and zones of water flow; no uniform, thick deposits observed.
- Adhesion /minor damage: most of the coating remained intact; slight discolouration and minor localised delamination were observed.
- Possible degradation mechanism: typical biofouling combined with the accumulation of dust and mineral deposits; surface degradation without significant structural damage.
- ER1 + ME coating—poor condition; poorest visible performance.
- Surface appearance: extensive areas covered with grey and black deposits; locally matte, with loss of gloss and colour changes compared with the original appearance.
- Biocorrosion: clearly developed biofilm; presence of algae visible as grey discolouration, indicating mixed organic–mineral deposits (biofilm combined with dust, soot, and microbial metabolic products).
- Adhesion/cracking: local delamination of the coating layer, microcracks, and isolated discolouration in areas of exposed concrete; adhesion reduced in zones with the highest deposit accumulation.
- Possible degradation mechanism: microbial colonisation by algae and bacteria may lead to biofilm formation and the retention of moisture and contaminants; microbial metabolites and matrix components (e.g., organic acids and extracellular polymeric substances [EPS]) may promote local surface degradation and accelerate combined chemical and mechanical deterioration of the coating.
- Potential consequence: further deterioration could reduce coating continuity, increase delamination, and facilitate water ingress into the concrete; reinforcement corrosion was not evaluated in this study.

**Figure 18:**
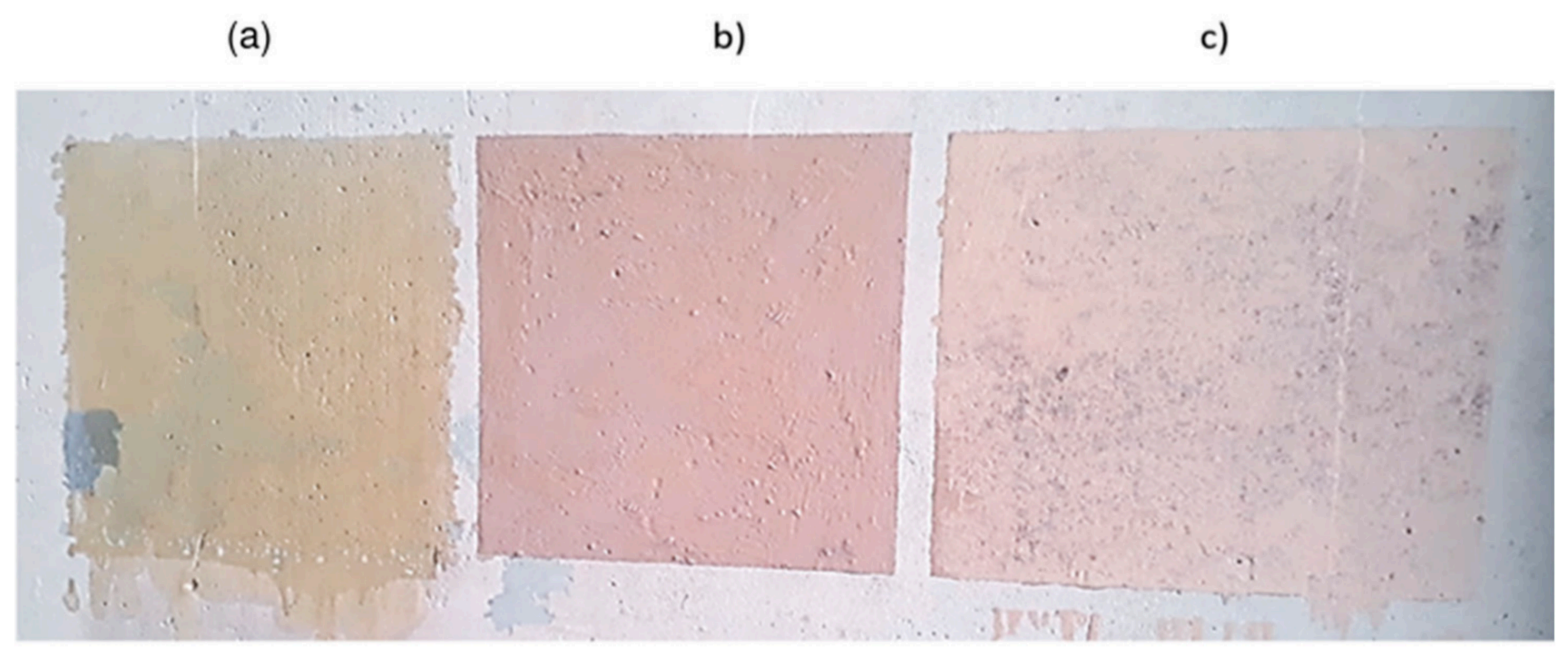
Reinforced concrete cooling-tower wall after one year of operation, following cleaning: (**a**) ER2 + ME, (**b**) ER1 + SA, (**c**) ER1 + ME.

Subsequently, the walls were cleaned using a high-pressure washer. The condition of the coatings after cleaning is presented in Figure 18.

A detailed description of the condition of the coatings after cleaning is presented below:

(a), (b) ER2 + ME and ER1 + SA coatings—very good condition

- Response to cleaning: high-pressure washing effectively removed dirt and biofilm from these surfaces. No noticeable change in the visible structure of the coating layer was observed, and both the aesthetic and protective were preserved.
- Surface appearance: uniform colour, no visible discolouration, with retained gloss and smoothness. No residual dirt or persistent deposits were observed on the surface.
- Interpretation: the limited residual contamination and biological colonisation are consistent with a relatively continuous surface and low contaminant adhesion; coating porosity and permeability were not quantified after field exposure. The material exhibits hydrophobic properties and maintains the barrier function of the coating, resulting in high aesthetic and technical durability.

(c) ER1 + ME coating—unsatisfactory condition

- Response to cleaning: despite intensive high-pressure washing, the surface did not recover its original appearance. The coating appeared matte, suggesting damage to the surface layer and a loss of smoothness.
- Surface appearance: residual contamination and persistent dark deposits remained visible on the surface. Even after cleaning, the aesthetic quality of the coating was significantly inferior compared to ER2 + ME and ER1 + SA.
- Material characteristics: the coating surface appeared relatively porous and irregular, which may promote the retention of contaminants, microbial metabolic products, and mineral deposits. This visible surface roughness also facilitates rapid recolonisation by algae and bacteria.
- Consequences: the loss of smoothness and surface gloss leads to increased water retention and facilitates the adhesion of new contaminants. In the long term, this may result in further aesthetic degradation as well as a reduction in the protective performance of the coating.

ER2 + ME and ER1 + SA showed favourable visible conditions and cleanability after washing under the specific cooling-tower exposure. By contrast, ER1 + ME retained deposits and showed surface deterioration. Contamination indicate that this material is less suitable for application in the cooling tower environment, characterised by high humidity, biofilm formation, and intensive atmospheric deposition. In the long term, the ER1 coating modified with methyl esters is likely to require more frequent maintenance in high-humidity replacement with a system exhibiting improved hydrophobic and biological resistance.

The detailed physicochemical mechanisms governing the modification of the ER1 and ER2 epoxy systems with ME and SA were presented in the preceding complementary study [31] and are therefore only summarised here. ME is a low-viscosity liquid modifier, whereas SA is crystalline at room temperature and requires melting before incorporation. In the previous study, ME reduced the viscosity of ER1 by more than 70%, compared with an approximately 50% reduction produced by SA. The liquid form and lower viscosity of ME facilitated its homogeneous distribution during sonication, improved substrate wetting, and enabled the resin to fill surface irregularities more effectively.

As proposed in [31], the long aliphatic residues of ME become distributed within and may be physically immobilised in the cross-linked epoxy network during curing. Their non-polar hydrocarbon chains reduce water affinity, while the cured epoxy phase provides a cohesive surface barrier. The favourable field condition of ER2 + ME may therefore be associated with the combined effects of homogeneous modifier distribution, reduced wettability, resistance to moisture transport, and stability under cyclic environmental exposure. This interpretation is consistent with the previously reported water-penetration, freeze–thaw, and salt-crystallisation results [31].

SA provided higher initial hydrophobicity in selected systems, whereas ME offered technological advantages associated with its liquid form and lower viscosity. Accordingly, the one-year field observations should not be interpreted as demonstrating the universal superiority of ME over SA but as evidence that field preservation depends on a combination of hydrophobicity, coating homogeneity, moisture resistance, and polymer-network stability. Coating–substrate adhesion was not quantified in either study and will be evaluated in a separate experimental programme.

The surface parameters investigated in the present study constitute one component of a broader durability assessment of the developed protection systems. Direct durability-related measurements for the same concrete composition and corresponding coating formulations were reported in the preceding complementary study [31]. These measurements included water absorptivity, water penetration under pressure, water-vapour diffusion, freeze–thaw resistance, and sulphate-salt crystallisation resistance.

In Reference [31], selected modified epoxy systems reduced water absorptivity by approximately 82% relative to the untreated concrete. The mean depth of water penetration decreased from 13.76 cm for the reference concrete to 1.57 cm for ER1 + ME, 1.72 cm for ER1 + SA, and 1.76 cm for ER1. The coated concrete achieved watertightness class W8, with no water seepage at a maximum pressure of 0.8 MPa. After 50 freeze–thaw cycles, mass changes of only 0.01% and 0.02% were recorded for ER2 + ME and ER1 + ME, respectively, compared with 3.11% for the untreated concrete. The coatings also substantially reduced deterioration during sulphate-salt crystallisation, whereas the reference concrete exhibited a mass loss exceeding 15%.

The apparent static water contact angle, surface free energy, roughness, and plan-view morphology examined in the present study provide complementary information on the surface conditions associated with this previously demonstrated macroscopic performance. They should not, however, be interpreted as direct measurements of chloride ingress, carbonation, coating adhesion, or reinforcement corrosion. The one-year cooling-tower exposure additionally demonstrates the visible field condition, contamination resistance, and cleanability of selected systems under actual service conditions but does not constitute an electrochemical assessment of reinforcement protection.

The field observations indicate that degradation inside the cooling tower was governed primarily by moisture-related and biological processes rather than by direct UV radiation, precipitation, or chloride exposure. Prolonged wetting, condensation, water runoff, and high humidity promoted biofilm development and the retention of organic and mineral deposits. Repeated wetting–drying and seasonal temperature changes may additionally have generated local stresses in the coating–substrate system.

ER2 + ME exhibited the most favourable visible condition after one year under these specific water-dominated exposure conditions. This result should not be extrapolated directly to marine, chloride-contaminated, highly irradiated, or strongly freeze–thaw environments. Dedicated controlled exposure tests would be required to compare performance under those conditions.

## 4. Conclusions

The main conclusions derived from this study are as follows:

- The apparent static water contact angle (CA) of the reference concrete was approximately 11°, confirming its hydrophilic character. All unmodified commercial resins prior to modification achieved CA values above 90°. The methyl-ester-modified systems showed CA values ranging from 91° to 127°, while ER1 + SA had the highest observed mean CA, at 131°. UV ageing produced small numerical decreases in CA for most systems.
- Fouling resistance tests demonstrated that ER1-based resins exhibited the greatest resistance to coal dust, both before and after modification with fatty-acid-derived additives. By contrast, the silane (S) and silicic acid ester (E) resins did not effectively protect the surface against ink contamination, even after modification. MR + SA coating exhibited hydrophobic behaviour, but residual stains could not be removed completely. ER1 + SA, ER1 + ME, and ER2 + ME showed the greatest stain resistance: the ink ran off the surface, and any remaining residue was readily removed.
- The average surface roughness of the ER1 coating decreased to R_a_ = 0.22 µm, which is approximately 24.5 times lower than that of the reference concrete. Resin modification reduced surface roughness and may therefore limit the retention of water and contaminants; it does not independently demonstrate improved corrosion protection or durability.
- A strong relationship was obtained between the apparent static water contact angles measured in the present study and the previously published water absorptivity values for the same protection systems (R^2^ = 0.9641) [31]. This secondary analysis indicates an association between surface wettability and macroscopic water uptake; however, it should not be interpreted as an independent validation dataset. Within the investigated set of chemically different protection systems, lower profile roughness was associated with higher apparent static water contact angles. This relationship reflects the combined effects of coating chemistry, surface coverage, porosity, and topography and should not be interpreted as a universal effect of roughness alone.
- Differences in coating microstructure arise from the chemical nature of the modifiers. Stearic acid (SA) due to its long hydrocarbon chain and polar -COOH group, interacts strongly with the epoxy matrix and imparts pronounced hydrophobic properties, whereas liquid methyl esters (MEs) were more readily dispersed and reduced resin viscosity. Both modifiers contain long hydrocarbon chains that may reduce surface affinity for water, but their molecular interactions with the cured resin were not determined in this study.
- After one year of operation, ER2 + ME coating exhibited the most favourable visible condition among the three systems assessed in situ. Only minor surface deposits and isolated stains were observed, while maintaining uniform coverage and a colour similar to its initial appearance. This indicates high resistance to environmental exposure and limited susceptibility to contamination. The coating maintained structural integrity, confirming good compatibility with the substrate and resistance to environmental factors such as moisture, temperature fluctuations, and biological activity. Following cleaning with a pressure washing, both ER2 + ME and ER1 + SA coatings retained their functional and aesthetic properties, demonstrating high resistance to environmental exposure and good cleanability without performance loss. These qualitative observations support their potential for reinforced-concrete structures exposed to biocorrosion and atmospheric conditions.

## Figures and Tables

**Table 1:** Characteristics of protective products used in the study.

Symbol	Product (Manufacturer, City, Country)	Type	Application	Composition/Ingredients	Density	Viscosity	Selected Properties
S	Nanobauer (Nanobauer Sp. z o.o., Wieliczka, Poland)	Organofunctional silane, solvent-based	Hydrophobic protection of concrete	Organofunctional silanes < 20%, KOH < 1%	~1.04 g/cm^3^ (20 °C)	—	Colourless, not water-miscible, used undiluted
ER1	Belzona 5811 + 4911 primer (Belse, Harrogate, UK)	Two-component epoxy (5:1)	Protection of concrete, reinforced concrete, and steel	Epoxy base + amine hardener	Mixed: 1.50–1.54 g/cm^3^	10,160 mPa·s (25 °C)	Sag > 375 µm, VOC 1.64%, adhesion 5.6 MPa (7 d), 34.8 MPa (28 d)
ER2	Belzona 4151 (Belse, Harrogate, UK)	Two-component solvent-free epoxy (2:1)	Sealing of concrete	Solvent-free epoxy system	Base: 1.16 g/cm^3^	500–700 mPa·s (25 °C)	Flexural 89.63 MPa, compressive 89.63 MPa, tensile 31.71 MPa, Shore 82, adhesion 3.45 MPa
E	Protectosil SH100 (Evonik Industries AG, Rheinfelden, Germany)	Silicic acid ester-based, solvent-free	Hydrophobic protection of concrete, stone, and brick	Tetraethylsilicate 10–25%, dioctyltin dilaurate 1–2.5%	~1.06 g/cm^3^ (25 °C)	~5 mPa·s (20 °C)	SiO_2_ 40–42 wt.%, flash point 43 °C, active > 98%
MR	Sikagard 680S + 700S primer (Sika Services AG, Baar, Switzerland)	One-component methacrylic resin, solvent-based	Protective and hydrophobic coating for concrete, reinforced concrete, and prestressed concrete	Aromatic solvent naphtha (40–60%), C9–C10 hydrocarbons (10–20%), alcohols and esters (<5%)	~1.4 kg/dm^3^	>20.5 mm^2^/s (40 °C)	Solids ~45%, DFT 101–290 μm, pull-off ≥ 2.0 MPa (≥1.5 MPa after F–T), capillary absorption ≤ 0.1 kg/(m^2^·h^0.5^), CO_2_ permeability ≥ 50 m, vapour ≤ 4 m, flash point ~25 °C

**Table 2:** Oxide composition of selected concrete specimens.

	E	E + ME	ER1 + SA	W
Compound	Oxide Content [%]			
SiO_2_	63.92	68.81	62.08	69.57
CaO	15.44	10.33	14.81	12.33
Al_2_O_3_	5.07	4.56	4.97	5.58
Fe_2_O_3_	2.54	2.60	3.16	2.53
K_2_O	1.47	1.37	1.72	1.84
SO_3_	0.55	0.53	1.37	0.61
TiO_2_	0.38	0.50	0.45	0.30
P_2_O_5_	0.22	0.13	0.01	0.52
MgO	0.09	0.01	0.31	0.41
MnO	0.04	0.01	0.02	0.03
LOI	10.28	11.15	11.10	6.28

**Table 3:** Results of the Kruskal–Wallis test for CA.

Variable	n	Statistic	df	*p*	Method
Apparent Static Water	48	43.175	15	<0.0001	Kruskal–Wallis

**Table 4:** Results of the Kruskal–Wallis test for CA after UV ageing.

.y. Variable	n	Statistic	df	*p*	Method
CA_after_UV	48	43.598	15	<0.001	Kruskal–Wallis

## Data Availability

The datasets generated and/or analysed during the current study are not publicly available, but are available from the corresponding author on reasonable request.
